# Nano-scale imaging of dual stable isotope labeled oxaliplatin in human colon cancer cells reveals the nucleolus as a putative node for therapeutic effect†

**DOI:** 10.1039/d0na00685h

**Published:** 2020-11-26

**Authors:** Anton A. Legin, Arno Schintlmeister, Nadine S. Sommerfeld, Margret Eckhard, Sarah Theiner, Siegfried Reipert, Daniel Strohhofer, Michael A. Jakupec, Mathea S. Galanski, Michael Wagner, Bernhard K. Keppler

**Affiliations:** Institute of Inorganic Chemistry, Faculty of Chemistry, University of Vienna A-1090 Vienna Austria anton.legin@univie.ac.at +43 1 4277 852601 +43 1 4277 52610; Research Cluster “Translational Cancer Therapy Research”, University of Vienna A-1090 Vienna Austria; Research Network “Chemistry Meets Microbiology and Environmental Systems Science”, University of Vienna A-1090 Vienna Austria; Division of Microbial Ecology, Large-Instrument Facility for Environmental and Isotope Mass Spectrometry, Centre for Microbiology and Environmental Systems Science, University of Vienna A-1090 Vienna Austria; Core Facility Cell Imaging and Ultrastructural Research, University of Vienna A-1090 Vienna Austria

## Abstract

Oxaliplatin shows a superior clinical activity in colorectal cancer compared to cisplatin. Nevertheless, the knowledge about its cellular distribution and the mechanisms responsible for the different range of oxaliplatin-responsive tumors is far from complete. In this study, we combined highly sensitive element specific and isotope selective imaging by nanometer-scale secondary ion mass spectrometry (NanoSIMS) with transmission electron microscopy to investigate the subcellular accumulation of oxaliplatin in three human colon cancer cell lines (SW480, HCT116 wt, HCT116 OxR). Oxaliplatin bearing dual stable isotope labeled moieties, *i.e.*^2^H-labeled diaminocyclohexane (DACH) and ^13^C-labeled oxalate, were applied for comparative analysis of the subcellular distribution patterns of the central metal and the ligands. In all the investigated cell lines, oxaliplatin was found to have a pronounced tendency for cytoplasmic aggregation in single membrane bound organelles, presumably related to various stages of the endocytic pathway. Moreover, nuclear structures, heterochromatin and in particular nucleoli, were affected by platinum-drug exposure. In order to explore the consequences of oxaliplatin resistance, subcellular drug distribution patterns were investigated in a pair of isogenic malignant cell lines with distinct levels of drug sensitivity (HCT116 wt and HCT116 OxR, the latter with acquired resistance to oxaliplatin). The subcellular platinum distribution was found to be similar in both cell lines, with only slightly higher accumulation in the sensitive HCT116 wt cells which is inconsistent with the resistance factor of more than 20-fold. Instead, the isotopic analysis revealed a disproportionally high accumulation of the oxalate ligand in the resistant cell line.

## Abstract 2

Correlated imaging by means of NanoSIMS and electron microscopy reveals the distribution of stable isotope labeled oxaliplatin in cancer cells.

## Graphical abstract

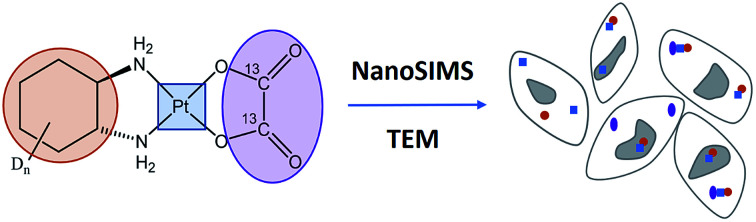

## Introduction

**Fig. 1:**
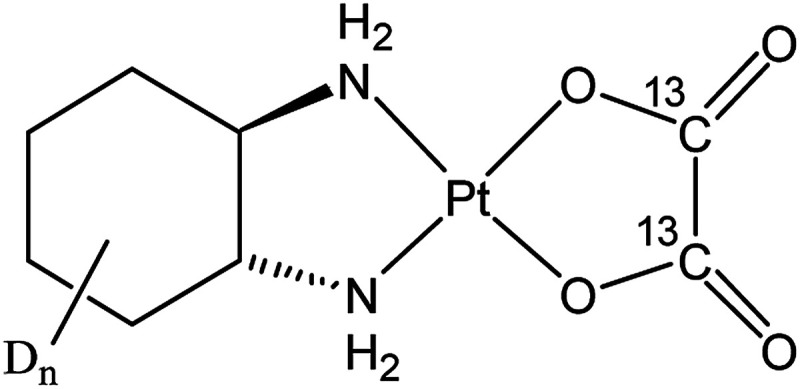
Structural formula of the applied dual stable isotope labeled oxaliplatin. In this study, an enantiomeric mixture containing *R*, *R*- and *S*, *S*- diaminocyclohexane (DACH) was synthesized. *D*_*n*_ refers to the number of hydrogen atoms of the cyclohexane ring substituted by deuterium (*n* = 10). From here on, the designation “oxaliplatin” will refer to the isotopically labeled compound, unless otherwise stated.

The third-generation platinum(ii) drug oxaliplatin, (*trans*-1*R*,2*R*-cyclohexane-1,2-diamine)oxalatoplatinum(ii) (Fig. 1), overcame some drawbacks of its predecessors, cisplatin and carboplatin. In particular, oxaliplatin proved to be active in a variety of *cis*-/carboplatin-resistant cell lines and tumors.^1^ The combination of oxaliplatin with 5-fluorouracil and leucovorin has emerged as the standard treatment for metastatic colorectal cancer, which is the second most frequent cause of cancer-related mortality in the developed countries.^2,3^ The main adverse side effect of oxaliplatin is its dose-limiting sensory neuropathy.^4^ Similar to cisplatin,^5,6^ the main target of oxaliplatin is supposed to be nuclear DNA with which it forms intra- and interstrand cross-links, with the diaminocyclohexane ligand protruding into the major groove. However, the cellular accumulation pattern and the reasons for the different range of oxaliplatin-responsive tumors as well as the weak cross-resistance between cisplatin and carboplatin remain incompletely explored. Recently, nucleolar and ribosomal stress were recognized as main responses to oxaliplatin treatment in cancer cells, which might explain the observed shift in the tumor selectivity of the drug.^7,8^

One of the fundamental strategies in exploring the activity/resistance mechanisms of chemotherapeutics is studying their distribution in the target tissues and cells. Suitable bioimaging techniques have evolved in the last decades, providing tools to follow the drug distribution on a subcellular scale.^9^ In particular, nanometer-scale secondary ion mass spectrometry (NanoSIMS) has paved the way for spatially resolved (below 100 nm) trace element and high-sensitivity isotope analysis and has proved its potential in subcellular distribution studies of metal-based drugs.^10–16^ SIMS can simultaneously yield information about the distribution of the central atom (*e.g.* Pt) and ligands containing isotopic tags such as ^2^H, ^13^C, ^15^N and ^18^O. Secondary ion signal intensity distribution images of biologically abundant elements (in particular nitrogen, phosphorus and sulfur) can readily be used to distinguish certain subcellular structures (*e.g.*, cell borders, nuclei, nucleoli). However, the exact identification of tiny organelles is highly challenging and thus requires application of imaging techniques capable of cellular ultra-structure characterization. The best means for simultaneous identification of distinct cellular compartments without organelle-specific labeling are electron microscopy (EM) based techniques. There are multiple examples of EM combined with SIMS for applications in microbiology,^17–19^ cell biology^20–22^ and in the field of chemical pharmacology.^23,24^ Here, we report the correlative application of NanoSIMS and transmission electron microscopy (TEM) for the subcellular investigation of isotopically dual labeled oxaliplatin in three human colon cancer cell lines (SW480, HCT116 wt and isogenic oxaliplatin resistant HCT116 OxR). High-pressure freezing followed by rapid, agitation-assisted freeze substitution and resin embedding was employed both for preservation of the native cellular structure and drug distribution.

For the first time, to our knowledge, the ultrastructurally correlated subcellular distribution of the clinically established platinum-based drug oxaliplatin in tumor cells is presented. Oxaliplatin showed a high affinity to cytoplasmic and nuclear structures similar to sites involved in cisplatin accumulation.^14^ We highlight the nucleolus as a target for interaction with platinum-based chemotherapeutics, as it is prone to platinum accumulation and/or decomposition in all tested settings.

## Materials and methods

### Chemicals and general procedures

All solvents and chemicals were purchased from commercial suppliers and were used without further purification. Isotopically labelled cyclohexanol-D_12_ was obtained from euriso-top. Thin layer chromatography was carried out on silica gel on TLC-PET foils (Fluka) and silica gel 60 (Fluka) was applied for column chromatography. Reactions involving platinum complexes were carried out under light protection using glass coated magnetic stirring bars. NMR measurements were recorded on a Bruker 500 MHz Avance III spectrometer at 500.32 (^1^H), 125.81 (^13^C) and 107.55 (^195^Pt) MHz in DMSO-d_6_ and D_2_O at 298 K. The solvent resonances were used as internal reference for ^1^H and ^13^C chemical shifts. ^195^Pt spectra were externally referenced to K_2_PtCl_4_. Electrospray ionisation (ESI) mass spectra were recorded on a Bruker amaZon SL ion trap mass spectrometer in positive and/or negative mode by direct infusion. High-resolution mass spectra were measured on a Bruker maXis™ UHR ESI time of flight mass spectrometer.

### Synthesis of (*SP*-4-2)-[*trans*-(D_10_)cyclohexane-1,2-diamine][(^13^C_2_)oxalato]platinum(ii)

(*SP*-4-2)-Dichlorido[*trans*-(D_10_)cyclohexane-1,2-diamine]platinum(ii) was synthesised from D_10_-cyclohexene according to literature (pathway 1).^25^ A mixture of (*SP*-4-2)-dichlorido[*trans*-(D_10_)cyclohexane-1,2-diamine]platinum(ii) (300 mg, 0.77 mmol, 1 eq.) and silver nitrate (254 mg, 1.5 mmol, 1.95 eq.) in water (10 ml) was stirred at room temperature for 8 h. Silver chloride was filtered off and sodium (^13^C_2_)oxalate (102 mg, 0.74 mmol, 0.95 eq.) was added to the clear solution. After stirring at room temperature for 24 h, the solution was concentrated *in vacuo* and cooled to 7 °C for 2 h. The precipitate was filtered off, washed with cold water and diethylether and dried *in vacuo*. The doubly labeled complex was yielded as a white solid. Yield: 260 mg (86%). ^1^H-NMR (500 MHz, DMSO-d_6_): *δ* = 6.09 (d, 2H, ^2^*J*_H,H_ = 9 Hz, N*H*), 5.36 (d, 2H, ^2^*J*_H,H_ = 9 Hz, N*H*) ppm. ^195^Pt-NMR (107 MHz, DMSO-d_6_): *δ* = −371 ppm. HR-MS [ESI(+), MeCN]: *m*/*z* = 430.1124 [M + Na^+^]^+^.

### Cell cultures and growth conditions

Three human colorectal carcinoma cell lines were used for the studies, *viz*. SW480 and an isogenic pair of HCT116 cell lines (the parental or wild type line and a subline with acquired resistance to oxaliplatin, resistance factor >20-fold). All cells lines were kindly provided by the Institute of Cancer Research, Department of Medicine I, Medical University of Vienna, Austria, authenticated and confirmed to be mycoplasma and cross-contamination free (Multiplexion, Germany, 2014). All cell lines were grown in RPMI 1640 medium (Sigma Aldrich) supplemented with 10% fetal bovine serum (Biowest) and 4 mM l-glutamine (Sigma Aldrich), referred to as complete RPMI 1640 medium below. Monolayer cell cultures were grown in 75 cm^2^ flasks (Starlab) at 37 °C in a moist atmosphere containing 5% CO_2_ in air.

### Fluorescence microscopy

SW480 cells were cultured on 18 × 18 mm glass cover slips placed in six-well plates (Starlab) at densities of 1.5 × 10^5^ cells per well in 1 ml of complete RPMI 1640 medium. The cells were allowed to attach for 24 h at 37 °C prior to drug exposure. After the treatment the cells were gently washed with PBS (3×) and stained with CytoPainter Nucleolar Kit (Abcam, ab139475) for 30 min according to the manufacturer's instructions (1 μl/1 ml 1× assay buffer, in the dark at 37 °C). The green fluorescence of nucleoli was detected under blue light excitation with a BX40 fluorescence microscope with an F-View CCD Camera, and 60× magnification oil immersion objective lens (Olympus). The resulting pictures were analyzed in Cell^F fluorescence imaging software where the size of nuclei/nucleoli was estimated according to the measured square (μm^2^). The treated samples were always compared relative to untreated control subjected to the same procedure.

### High-pressure freezing and resin embedding

ACLAR® discs (5 mm in diameter for fitting into the carriers for HPF) were punched out from a 1 μm thick ACLAR® fluoropolymer film (Science Services) and placed in 12-well plates. Monolayer cell cultures (SW480, HCT116 wt, HCT116 OxR) were seeded at densities of 2 × 10^5^ cells per well. They were allowed to settle for 24 h on ACLAR®. After cells had reached 80–90% confluence, they were incubated with freshly prepared oxaliplatin/cisplatin solutions (24 h, 50–200 μM) in complete RPMI 1640 medium. After drug treatment, cells were washed with RPMI 1640 medium supplemented with 20% FBS.

The ACLAR® discs were placed into 1-hexadecene (Merck) coated carriers type A (6 mm in diameter, 200 μm in depth; Leica) and filled up with 10% BSA in PBS. Samples were sandwiched between carriers type A and the flat side of carrier type B (6 mm in diameter, 300 μm in depth) and high-pressure frozen in a HPM100 freezer (Leica). The frozen samples were then transferred under liquid nitrogen into 2 ml Sarstedt tubes filled with 1 ml frozen substitution medium, 1% OsO_4_ in dried acetone containing 0.5% glutaraldehyde (Science Services). Subsequently, the Sarstedt tubes were inserted in the tube holders of an agitation module^29^ (Cryomodultech e.U.) in the cryochamber of the freeze substitution (FS) system AFS2 (Leica), which was precooled to −140 °C in advance. FS took place within *ca.* 5 h at −85 °C under agitation overnight followed by gradual warm up of the samples to room temperature. After washing with acetone, the samples were infiltrated and embedded in low viscosity epoxy resin (Agar Scientific). The resin blocks were polymerized in the oven at 65 °C for 96 h. The ACLAR® discs were removed from the resin and the blocks were divided into parts by sawing.

### TEM and SIMS sample preparation

To obtain three consecutive ultra-thin sections (75 nm in thickness) the resin-embedded cell monolayers were cut by using an ultramicrotome (Ultracut S; Leica) with a diamond knife (Diatome). The first section was placed on a copper grid, counterstained with uranyl acetate and lead citrate, and imaged in a TEM Libra 120 (Zeiss) at 120 kV. Multi-aligned images were acquired by using a bottom stage digital camera, TRS (4 MP), and iTEM software (Olympus). The second and third consecutive section were each deposited onto antimony-doped silicon wafer platelets (7.1 × 7.1 × 0.75 mm; Active Business Company) for correlated metal and isotopic label analysis by NanoSIMS. A wrinkle-free attachment of the sections was achieved by transferring them with a Perfect Loop (Science Service) onto pre-warmed wafer platelets, heated on a hotplate at 80 °C.

### NanoSIMS analysis

NanoSIMS measurements were carried out on a NS 50L Cameca instrument (France) as described previously.^23^ Briefly, the detectors of the multicollection assembly were positioned to enable parallel detection of ^16^O^1^H^−^, ^12^C_2_^−^, ^12^C^14^N^−^, ^31^P^−^, ^34^S^−^ and ^195^Pt^−^ secondary ions for platinum distribution measurements and ^1^H^−^, ^2^H^−^, ^16^O^1^H^−^, ^16^O^2^H^−^, ^12^C_2_^−^, ^12^C^13^C^−^ and ^12^C^14^N^−^ for ligand distribution measurements. Prior to data acquisition, analysis areas were pre-conditioned *in situ* by rastering of a high-intensity, defocused Cs^+^ ion beam in the following sequence of high and extreme low ion impact energies (HE/16 keV and EXLIE/50 eV, respectively): HE at 100 pA beam current to a fluence of 5.0 × 10^14^ ions per cm^2^ (for non Au-coated samples) and 2.5 × 10^15^ ions per cm^2^ (for Au-coated samples); EXLIE at 400 pA beam current to a fluence of 5.0 × 10^16^ ions per cm^2^; HE to a fluence of 2.5 × 10^14^ ions per cm^2^. All data were acquired as multilayer image stacks obtained by sequential scanning of a finely focused Cs^+^ primary ion beam over areas between 32 × 32 and 46 × 46 μm^2^ with 512 × 512 pixel image resolution and approx. 80 nm physical resolution (probe size). The per-pixel dwell time of the primary ion beam was 10–15 ms. Between every image cycle, secondary ion beam drift was corrected by automatic beam centering and coaxial lens (“EOS”) voltage optimization (utilizing the ^12^C_2_^−^ signal as a reference) as well as automatic peak centering for each of the recorded secondary ion species. The total acquisition time ranged from 15 to 25 h per measurement.

### NanoSIMS image processing, numerical data evaluation and statistics

Image processing was done as described previously.^23^ Briefly, NanoSIMS image data were evaluated by using the WinImage software package (version 2.0.8) provided by Cameca. Prior to stack accumulation, the individual images were drift corrected. Secondary ion signal intensities were corrected for detector dead time on a per-pixel basis and quasi-simultaneous arrival (QSA) of C_2_^−^ and CN^−^ secondary ions on a per-ROI basis. The QSA correction was performed according to the formalism suggested by previous work,^26^ applying sensitivity factors of 1.06 and 1.05 for C_2_^−^ and CN^−^ ions, respectively (experimentally determined on dried yeast cells – data not shown). For H^−^ ions, a factor of 1.00 was chosen. ROI data were analyzed for normal distribution (Kolmogorov–Smirnov test, *p* < 0.05). Normally distributed data were tested for significant differences in the arithmetic means by application of Welch's *t*-test, not normally distributed datasets were tested for significant differences in the medians by application of the Mann–Whitney *U*-test. Statistical calculations were conducted with the GraphPad Prism software. TEM/NanoSIMS overlay images were generated utilizing the GIMP 2.10 software.

### NanoSIMS data representation

For comparison of SIMS data acquired in different measurement runs and/or on different samples, the secondary ion signal intensity associated with the analyte needs to be normalized to an appropriate reference signal. For organic materials such as biomass, carbon is abundant in the matrix which renders C^−^ or C_2_^−^ secondary ion beam intensities well suited as reference signals. Owing to its higher ion yield, we chose ^12^C_2_^−^ as the reference signal in the platinum measurements. However, ^13^C isotope labeling leads to variation of the ^12^C_2_^−^ signal intensity in relation to the label content, which introduces a systematic error in the inferred analyte to reference signal intensity ratios. In order to avoid biasing, we used the following expression for calculation of the normalized platinum signal intensities:1
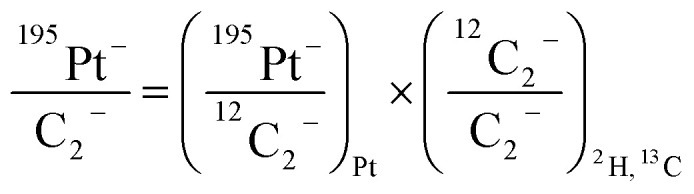
where ^195^Pt^−^/C_2_^−^ refers to the overall C_2_^−^ normalized ^195^Pt^−^ signal intensity, (^195^Pt^−^/^12^C_2_^−^)_Pt_ to the ^12^C_2_^−^ normalized ^195^Pt^−^ signal intensity determined in the platinum measurement. (^12^C_2_^−^/C_2_^−^)_^2^H,^13^C_ refers to the relative contribution of ^12^C_2_^−^ ions to the total C_2_^−^ signal intensity, which was determined in the isotope measurements *via* detection of ^12^C_2_^−^ and ^12^C^13^C_2_^−^ utilizing the expression2
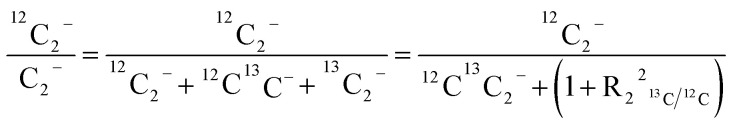
in which *R*_^13^C/^12^C_ refers to the ^13^C over ^12^C isotope ratio, given by *R*_^13^C^/12^C_ = ^12^C^13^C^−^/(2^12^C_2_^−^).

As demonstrated in a previous publication on ^15^N isotopically labeled cisplatin (14), variable transformation of isotope composition data enables inference of the relative elemental content of ligands to the total elemental content within the analyzed region. The transformation is based on mass balance calculations which, in a generalized notation for two ligands (L1 and L2), can be written as:

3 *n*_X,tot_ = *n*_X,L1_ + *n*_X,L2_ + *n*_X,*c*_ + *n*_X,*r*_

where *n* refers to the number of atoms of element X, contained in the sampled region (tot) consisting of contributions from the ligands (L1, L2), the cellular biomass (*c*) and the resin matrix (*r*). In an additional mass balance equation, the number of label atoms (^A^X) is considered:

4 *n*_^A^X,tot_ = *n*_^A^X,L1_+ *n*_^A^X,L2_ + *n*_^A^X,*c*_ + *n*_^A^X,*r*_

Utilizing the isotope fraction, which is, for an element containing two isotopes (^A^X, ^B^X), given by5
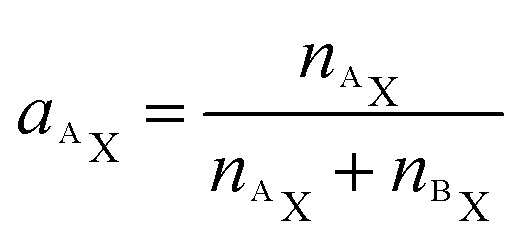
eqn (3) and (4) can be combined to6
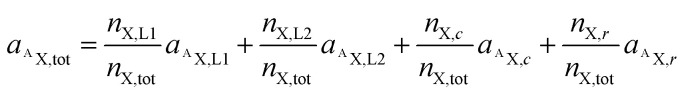


Let us now consider oxaliplatin and let L1 and L2 be the DACH (DA) and the oxalate (Ox) ligand, respectively. For oxalate, equ. (6) reads7
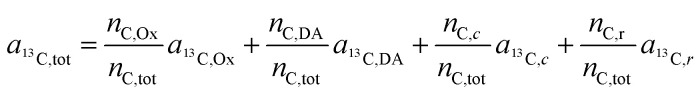


In the labeling experiments, the ^13^C/(^12^C + ^13^C) isotope fraction of oxalate (*a*_^13^C,Ox_) was 99 at%. The ^13^C content of unlabeled resin embedded cells was determined as 1.072 ± 0.001 at% (untreated SW480 cells, serving as control), the carbon atoms in the DACH ligand were isotopically unlabeled. It should be noted that, due to the lack of certified reference materials, NanoSIMS isotope composition measurement values are not absolute, and we applied the value obtained from the control sample on all compounds that were considered as unlabeled, *i.e.*

8 *a*_^13^C,DA_ = *a*_^13^C,*c*_ = *a*_^13^C,*r*_ = *a*_^13^C,ctrl_.

Accordingly, eqn (7) adapts9
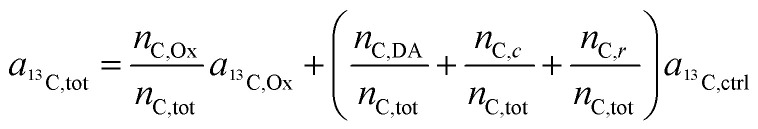
which, on account of the relationship displayed in the first mass balance equation (eqn (3)) can be further simplified to10
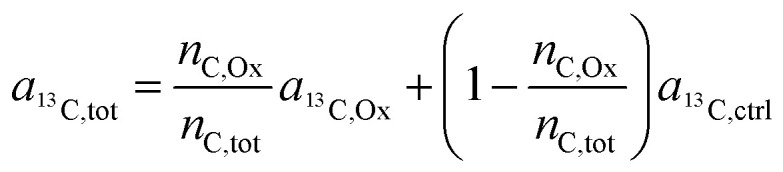


Finally, through rearrangement, an expression can be obtained that displays the number of carbon atoms originating from the oxalate ligand relative to the total carbon atoms contained in the sampled region:11
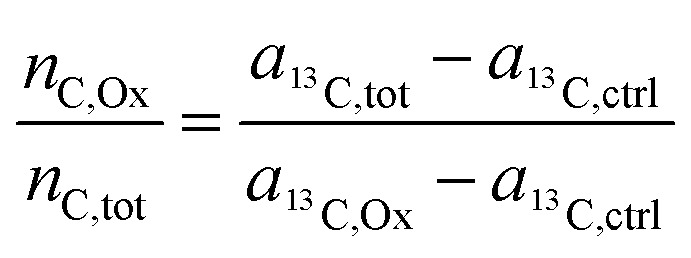


For the deuterium labeled DACH ligand (DA), equ. (6) can be written as:12
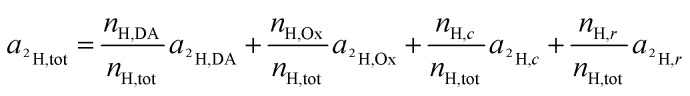


For platinum bound oxalate, *n*_H,Ox_/*n*_H,tot_ = 0, which leads to cancellation of the second term on the right side of eqn (12). Upon cleavage from the Pt center, oxalate gets partially protonated under physiological pH conditions. However, these protons originate from – isotopically unlabeled – water. In similarity to the consideration about ^13^C, the deuterium isotope fraction of all unlabeled compounds was assumed as being equal to the deuterium content measured on the untreated, resin embedded SW480 cells (0.011 ± 0.001 at%), *i.e.*

13 *a*_^2^H,Ox_ = *a*_^2^H,*c*_ = *a*_^2^H,*r*_ = *a*_^2^H,ctrl_

and eqn (12) simplifies to14
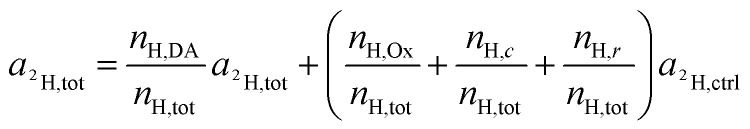


Again, according to the first mass balance equation, eqn (3) can be further simplified to15
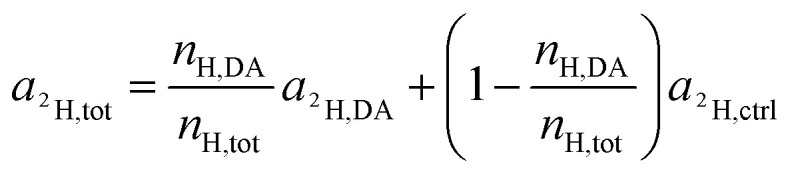


Rearrangement of this equation yields the expression which relates the number of hydrogen atoms originating from the DACH ligand to the total number of hydrogen atoms contained in the sampled region:16
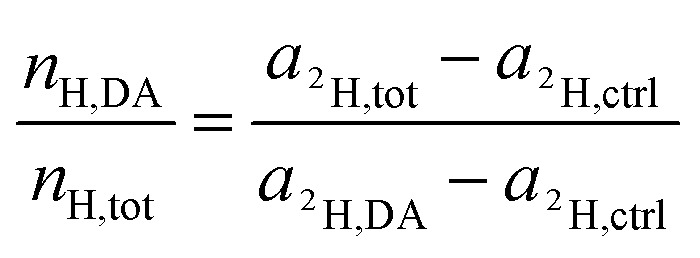


Fig. 7 and S7† show the values calculated from eqn (11) and eqn (16) plotted *versus* the C_2_^−^ and H^−^ normalized ^195^Pt^−^ normalized signal intensities. The normalized signals (displayed in arbitrary units) may be considered as being proportional to the platinum content relative to the total carbon or hydrogen content. Consequently, the slopes shown in the correlation plots refer to the ligand to central atom ratios of the accumulated drug.

### Determination of the total cellular platinum content by ICP-MS

Cellular uptake experiments were performed to determine the total cellular drug concentration. Cells (SW480, HCT116 wt, HCT116 OxR) were seeded into 6-well plates (Starlab) in densities of about 2.5 × 10^5^ cells per well. Cell microcultures were incubated in a moist atmosphere at 37 °C for 24 h prior to exposure to the drugs (cisplatin, oxaliplatin, both 50 μM for 24 h). The cell number was determined in a hemocytometer by using trypan blue staining in parallel microcultures during the 2 h exposure period. After exposure to the drugs, the medium was removed, cells were washed with PBS and consecutively lyzed with 0.5 ml sub-boiled HNO_3_ (conc.) per well for 1 h. Ultrapure water (resistivity ≥ 18.2 MΩ cm) from a Milli-Q Element water purification system and nitric acid (≥69%, TraceSELECT®, Fluka) were used for all dilutions for ICP-MS measurements. Elemental standard stock solutions of rhenium and platinum were obtained from CPI International. The total platinum content was determined with an Agilent 8800 ICP-MS/MS instrument (Agilent Technologies) equipped with nickel cones and a MicroMist nebulizer at a sample uptake rate of approximately 0.25 ml min^−1^. The following ICP-MS instrument settings were used: radio frequency power of 1550 W, a plasma Ar gas flow rate of 15 L min^−1^, a carrier Ar gas flow rate of ∼1.08 L min^−1^ and an auxiliary Ar gas flow rate of ∼0.90 L min^−1^. Quantification was performed by using the isotopes ^185^Re and ^195^Pt, which were monitored with integration times of 0.3 s, 10 sweeps per replicate and 10 replicates, whereas Re served as an internal standard for Pt. The Agilent MassHunter software package (Workstation Software 4.3, Version C.01.03, 2016) was used for data processing.

### Cell apoptosis assay

To investigate the cell viability and possible death induction by oxaliplatin, SW480 cells were analyzed by fluorescence-activated cell sorting (FACS) using fluorescein isothiocyanate (FITC)-conjugated annexin V (BioVision, USA) and propidium iodide (PI; Fluka) staining. SW480 cells were seeded into 6-well plates (CytoOne, Starlab, UK) in densities of 2 × 10^5^ cells per well in complete RPMI medium and allowed to settle for 24 h. The cells were exposed to different concentrations (50–100–200–400 μM) of oxaliplatin for 24–48 h at 37 °C. After incubation, the cells were gently trypsinised, collected in 2 ml tubes, washed with PBS and resuspended with FITC-conjugated annexin V (0.5 μg ml^−1^) in binding buffer (10 mM HEPES/NaOH pH 7.4, 140 mM NaCl, 2.5 mM CaCl_2_) at room temperature for 30 min. PI (2 μg ml^−1^) was added shortly before the measurement. Stained cells were analyzed with a Guava 8HT EasyCyte flow cytometer (Merck Millipore, USA) using InCyte software. Three independent experiments were conducted, at least 5000 cells were analyzed in each run.

## Results

In order to investigate the subcellular fate of the metal centerand the ligands, an oxaliplatin batch with isotopically distinctly labeled leaving and non-leaving groups was synthesized as racemic mixture and applied in all experiments (Fig. 1). (^13^C_2_)oxalate was obtained from a commercial supplier, whereas the diaminocyclohexane (DACH) ligand featuring deuterium labeling was synthesized starting from cyclohexanol-D_12_ (it should be noted that, in order to avoid isotope re-exchange in aqueous solution, only carbon-bound hydrogen atoms were substituted by deuterium).

Due to the high oxaliplatin concentrations used in the imaging experiments (*e.g.* 200 μM in SW480 cells) we tested the potency of the drug to induce the cell death with 24 h exposure time. With the annexin V – propidium iodide assay the viability of the cells was confirmed to stay above 90% at relevant concentrations (50–200 μM, 24 h exposure, Fig. S10†). The reason for such an effect might lay in a late onset for cytotoxicity of platinum(ii)-based chemotherapeutics as also shown previously for cisplatin^14^ (Fig. S9 and S10 in ESI of ref. 14†).

Owing to the high drug accumulation upon treatment with both cisplatin and oxaliplatin (Table 1), SW480 cells were used for method validation. For subcellular drug distribution studies, SW480 cell monolayers were grown on a thin polymer foil (ACLAR®), which allowed sustainment of the cells in the adherent state and cryo-immobilization by high-pressure freezing within milliseconds (see ref. 27 and section Materials and Methods for details). This approach promotes preservation of the cellular ultrastructure (*e.g.*, cytoskeleton, organelle networks, cellular compartmentalization and the close extracellular matrix) in the native condition and opens possibilities to minimize re-distribution or elution of the drug through the subsequent sample dehydration at low temperatures. By using an in-house established agitation module for sample agitation in an automated freeze substitution system (AFS), we accelerated the FS process and minimized the extraction of cellular material by organic solvents, compared to long-term FS or conventional chemical fixation, dehydration and embedding at room temperature.^28,29^

**Table 1:** Total cellular platinum accumulation (fg per cell) as determined by ICP-MS after 24 h drug exposure (50 μM)

	SW480	HCT116 wt	HCT116 OxR
Oxaliplatin^a^	27 ± 8	11 ± 1	8.5 ± 0.1
Cisplatin	59 ± 3	34 ± 7	27 ± 5

a Isotopically unlabeled oxaliplatin was applied in accumulation measurements.

**Fig. 2:**
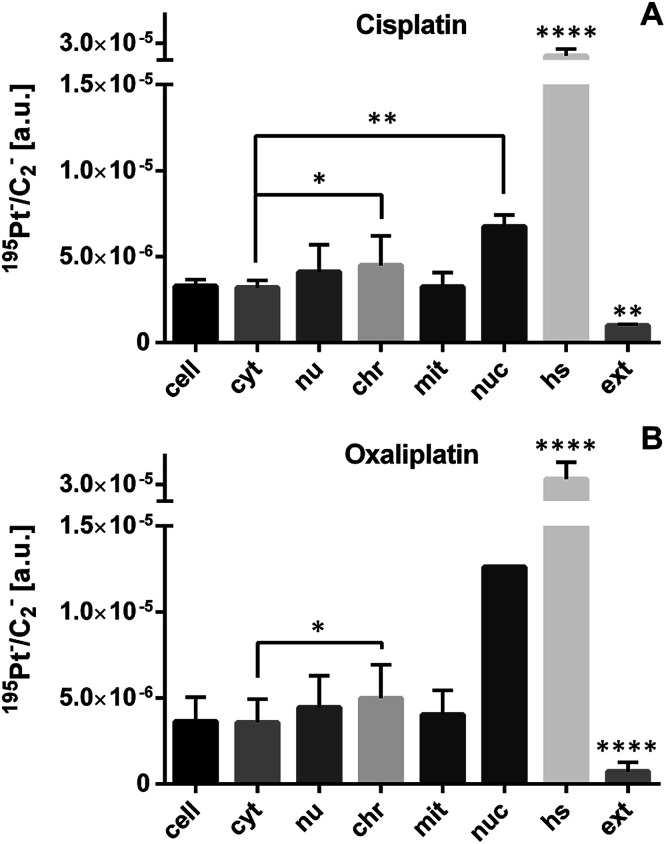
Sub-cellular platinum distribution in SW480 cells as determined by NanoSIMS. Cells of an adherent culture were exposed for 24 h to 50 μM cisplatin (A) or 200 μM oxaliplatin (B). The displayed values were obtained by ROI-specific evaluation of C_2_^−^ normalized ^195^Pt^−^ signal intensity distribution images. Both drugs exhibit preferential accumulation in cytoplasmic aggregations (hotspots) and in nuclear structures. Abbreviations and number of data points per ROI: cell – entire cell (*n* = 4 cisplatin, 11 oxaliplatin), cyt – cytoplasm (*n* = 4, 10); nu – nucleus (*n* = 4, 7); chr – chromatin (*n* = 13, 26); mit – mitochondria (*n* = 9, 32); nuc – nucleolus (*n* = 3, 1); hs – hotspots (*n* = 37, 118), ext – extracellular matrix (*n* = 8, 24). Data are presented as means ± SD. Statistical analysis: two-sided Student's *t*-test with Welch's correction (*p < 0.05; **p < 0.01; ****p < 0.0001). Note that only one nucleolus was accessible for NanoSIMS analysis from the oxaliplatin treated cells and the single measurement value was excluded from significance testing.

ICP-MS bulk analysis revealed a factor of two to three times higher cellular accumulation of cisplatin than for oxaliplatin (Table 1). Due to higher accumulation of both platinum-based drugs in SW480 cells, we selected this cell line for comparative analysis of the sub-cellular drug distribution (Fig. 2). Both drugs exhibited highly similar platinum distribution patterns, revealing cytoplasmic aggregation, pronounced affinity to nucleolar structures and a tendency for accumulation in chromatin, whereas no significant platinum accumulation in mitochondria was observed. These data are consistent with DNA as the generally accepted target for platinum drugs.^30–32^

### Oxaliplatin affects nucleoli in SW480

**Fig. 3:**
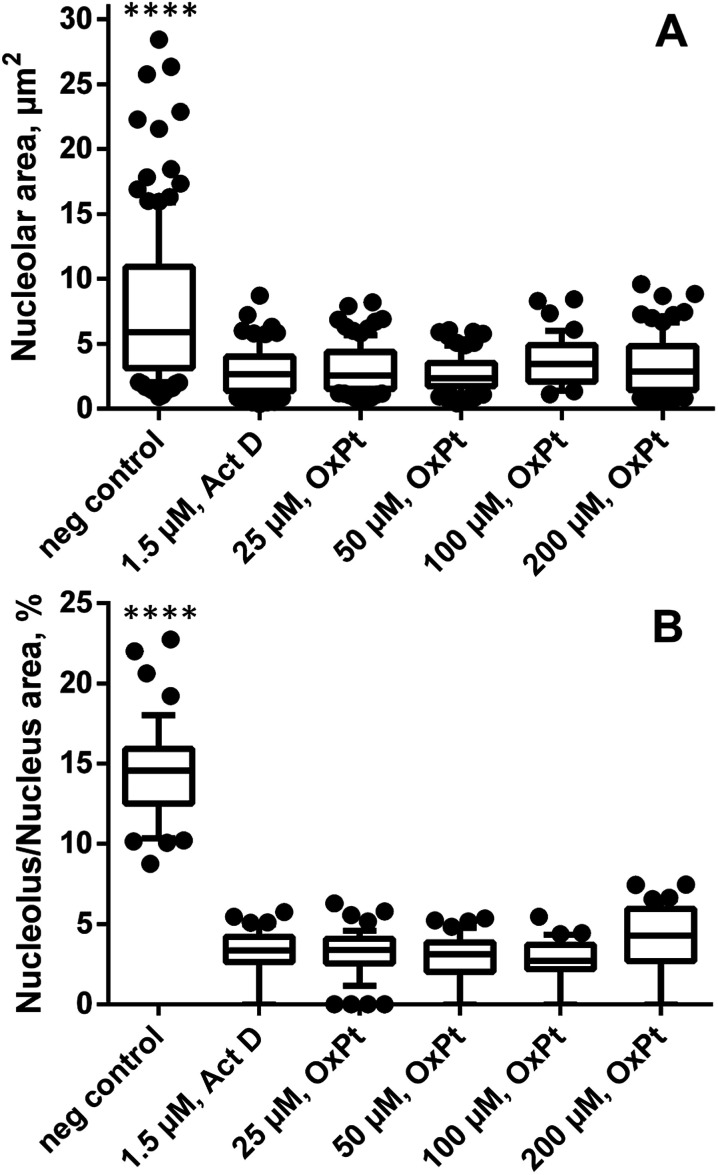
Oxaliplatin treatment affects nucleolar integrity in adherent SW480 cells. The graphs show the effect of oxaliplatin exposure (24 h, various concentrations) on the nucleolar absolute size (A) and fractional area within nuclei (B) as observed by fluorescence microscopy. Data are presented as box and whisker plots displaying the median, 25^th^, 75^th^, 10^th^ and 90^th^ percentiles. Abbreviations: neg control – negative control (untreated cells). Statistical analysis: Kolmogorov–Smirnov normality test, Mann–Whitney *U*-test (****p < 0.0001).

Remarkably, in oxaliplatin treated SW480 cell monolayers subjected to correlated TEM/NanoSIMS analysis, only a few intact nucleoli could be identified by TEM (*n*_cells_ > 30) and, according to the relative elemental composition, only one microscopically pre-defined nucleolus (*n*_cells_ = 10) was confirmed to exhibit the characteristic high levels of phosphorus, sulfur and nitrogen. Based on this observation, we hypothesized a possible interaction between oxaliplatin treatment and nucleolar integrity, which was further studied by means of fluorescence microscopy (Fig. 3 and S8†). The absolute size of nucleoli in cells treated with oxaliplatin in the concentration range from 25 to 200 μM, as well as actinomycin D (1.5 μM, positive control), was shown to be on average 2.5-fold smaller than in untreated cells (Fig. 3A). Moreover, the partial volume and abundance within nuclei was strongly altered: in all oxaliplatin treated samples, nucleoli occupied on average less than 5% (3.4 ± 0.7%) of the nuclear area (with some cells being completely devoid of detectable nucleoli), whereas, in untreated cells, nucleoli were highly abundant and occupied on average 15% (14.6 ± 3.1%) of the nuclear area (Fig. 3B). Morphologically, the effect of the platinum drug is similar to that of actinomycin D – a DNA-dependent RNA synthesis inhibitor widely used to induce segregation and distress of the nucleolus.^33,34^ In both cases the treatment results in significant reduction of both nucleolar number and size (Fig. S8†).

### Platinum distribution in SW480 cells

**Fig. 4:**
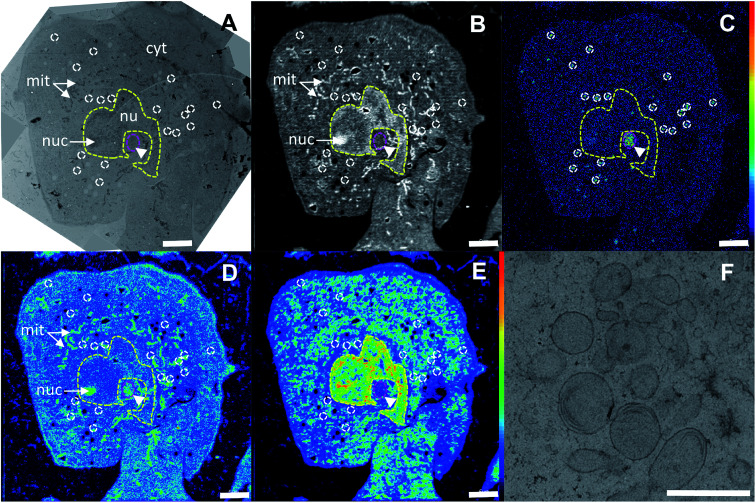
Subcellular distribution of oxaliplatin in a SW480 colon cancer cell (24 h, 200 μM). (A) – TEM survey image; (B) – ^12^C^14^N^−^ secondary ion map indicating the relative nitrogen distribution; (C) – ^195^Pt^−^ secondary ion map displaying the central atom distribution (hotspots are outlined in white); (D) – ^34^S^−^ secondary ion map highlighting rod-shaped mitochondria (arrows) and a perinuclear structure (arrowhead) rich in sulfur; (E) − ^31^P^−^ secondary ion map utilized for definition of the nuclear region (yellow outline). (F) – High-resolution TEM image of the single-membrane bound organelles showing lamellar inclusions associated with platinum accumulation within the perinuclear region (marked by the magenta ellipse region in (A–E)). Abbreviations: cyt – cytoplasm, nu – nucleus, nuc – nucleolus, mit – mitochondria. Intensities are displayed on a false-color scale ranging from low intensities (black/blue) to high intensities (white/red). Scale bars = 5 μm (A–E), 1 μm (F).

In the correlated TEM/NanoSIMS images of an oxaliplatin treated SW480 cell (Fig. 4, see Fig. S1† for the detailed alignment procedure), the subcellular structures can readily be identified. The ^12^C^14^N^−^ secondary ion map aided in definition of the cell boundaries, which were used for a basic spatial alignment of SIMS images with TEM micrographs (Fig. 4A and B). Chromatin structures anchored on the nuclear membrane and highlighted in the ^31^P^−^ ion map were used to define the nucleus (Fig. 4E, yellow dash). Within the nucleus, a sulfur and nitrogen rich structure, visualized by the ^34^S^−^ and ^12^C^14^N^−^ secondary ion signal intensity patterns, indicates a nucleolus. Pt rich cytoplasmic aggregates were selected (white outline in the ^195^Pt^−^ secondary ion map) and projected onto the TEM image to identify the structures associated with platinum accumulation (Fig. 4C and F). In SW480 cells, platinum was shown to aggregate in sulfur-rich single-membrane bound cytoplasmic organelles (Fig. S2A and B†) with some indication of lamellarization and vesiculation (Fig. 4F), similar to those associated with cisplatin accumulation in a parallel experiment (Fig. S3†). Presumably, these organelles belong to the endocytic and/or lysosomal pathway as reported previously for cisplatin.^14,35^ A moderate platinum signal intensity enhancement can also be observed in the nucleolar region of the nucleus. No colocalization of platinum hotspots with mitochondria, endoplasmic reticulum, lipid droplets or Golgi structures was observed. Overall, both drugs show very similar subcellular platinum distribution patterns (Fig. 2).

### Platinum distribution in HCT116 cells

**Fig. 5:**
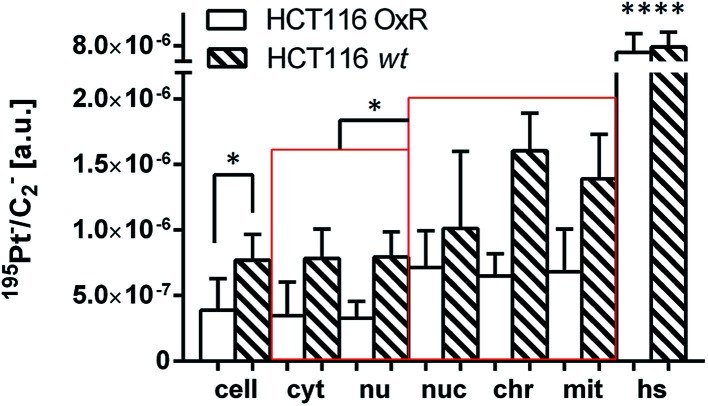
Subcellular platinum distribution in HCT116 wt and HCT116 OxR cells. The cells were exposed to 100 μM oxaliplatin for 24 h. The displayed values were obtained from ROI-specific evaluation of C_2_^−^ normalized ^195^Pt^−^ signal intensity distribution images. Note that in both HCT116 cell lines the local platinum concentrations within nucleolar, chromatin and mitochondrial compartments are significantly higher than the average concentrations within the cytoplasm/nuclei. Abbreviations and number of data points per ROI: cell – entire cell (*n* = 8 HCT116 OxR, 3 HCT116), cyt – cytoplasm (*n* = 7, 3); nu – nucleus (*n* = 8, 3); nuc – nucleolus (*n* = 9, 3); chr – chromatin (*n* = 14, 6); mit – mitochondria (*n* = 13, 8); hs – hotspots (*n* = 48, 19). Data are presented as means ± SD. Statistical analysis: two-sided Student's *t*-test with Welch's correction (*p < 0.05; ****p < 0.0001).

The next step was to investigate the fate of oxaliplatin in tumor cells differing in sensitivity to the drug. For this purpose, the isogenic colon cancer cell lines HCT116 wt and HCT116 OxR (with acquired resistance of >20-fold) were exposed to the drug for 24 h and subjected to correlative TEM/SIMS analysis. The total cellular and organelle-specific platinum accumulation was shown to be higher in HCT116 wt cells than in its resistant counterpart (Fig. 5, first two columns), which is in agreement with the result from the ICP-MS bulk analysis (Table 1). Interestingly, no pronounced difference between the sites of subcellular platinum accumulation was observed – both cell types tend to accumulate the drug in cytoplasmic aggregates, mitochondria and phosphorus-rich nuclear structures (chromatin, nucleoli).

**Fig. 6:**
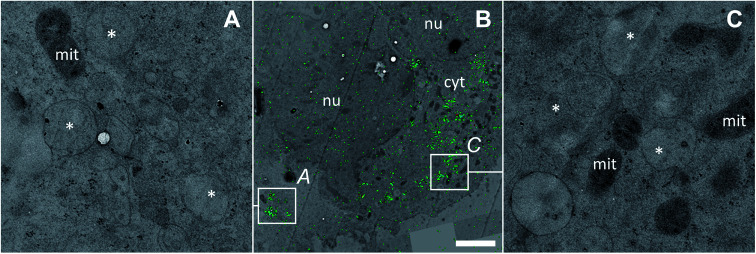
Subcellular platinum distribution in an adherent HCT116 OxR cell after 24 h exposure to 100 μM oxaliplatin. (A and C) – TEM micrographs showing single-membrane bound organelles with vesicular inclusions (asterisks) associated with cytoplasmic platinum accumulation; (B) – overlay of the NanoSIMS ^195^Pt^−^ secondary ion map (green) and the TEM image (black/white) visualizing the overall platinum distribution (green) within the cell. The colocalization is exemplified in Fig. S9.† Abbreviations: cyt – cytoplasm, mit – mitochondria, nu – nucleus. Scale bar = 3 μm.

Discrete single-membrane bound cytoplasmic organelles with characteristic lamellar and/or vesicular inclusions were identified as sites of platinum accumulation in HCT116 OxR (Fig. 6) and HCT116 wt (Fig. S4†) cells, morphologically similar to those in SW480 cells (Fig. 4F). Notably, in contrast to SW480 cells, these structures showed decreased intensities of the nitrogen and sulfur associated secondary ion signals (^12^C^14^N^−^ and ^34^S^−^, Fig. S5†). The apparently low protein and sulfur content lead us, in combination with the morphological appearance, to the assumption that the cytoplasmic organelles accumulating platinum in HCT116 cells may represent early endocytic vesicles rather than late endosomes or lysosomes as in the case of SW480 cells.

### Ligand distribution patterns

**Fig. 7:**
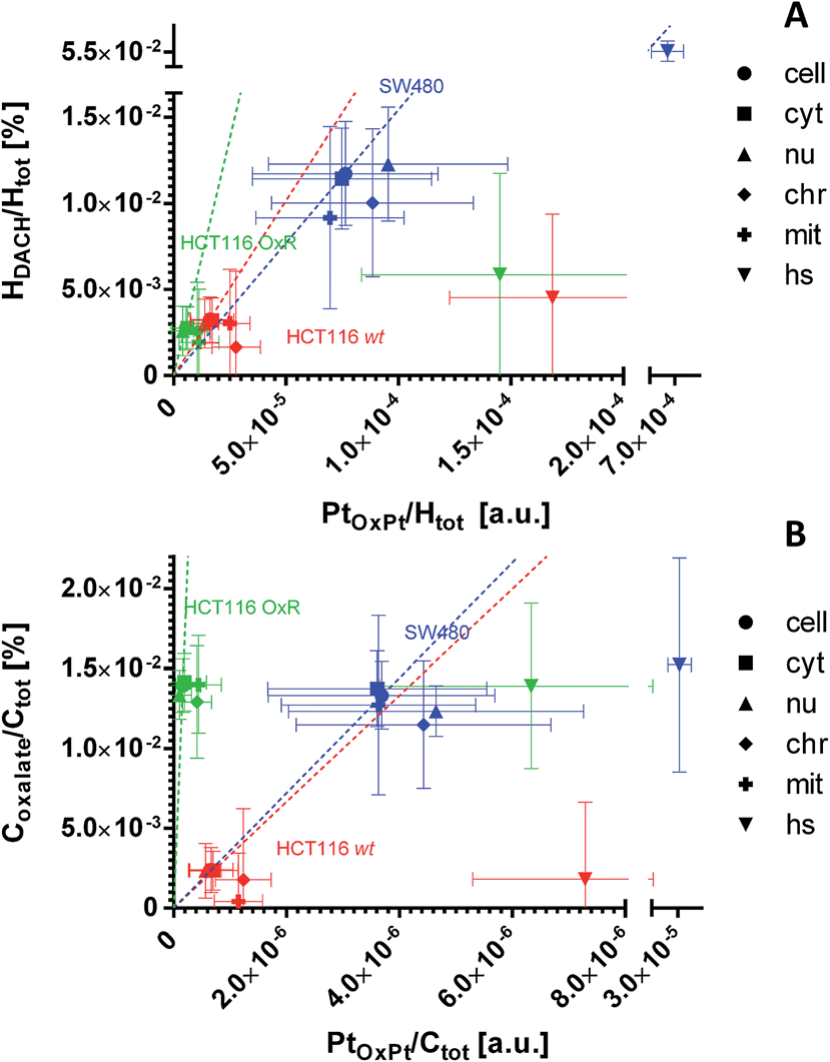
Subcellular distribution of the ligands relative to platinum as inferred from NanoSIMS elemental mapping and determination of the local hydrogen and carbon isotope compositions. The distribution of the DACH ligand (non-leaving group) relative to the platinum (A) shows an almost linear relationship for the major cellular compartments of all 3 cell lines (*e.g.* cytoplasm, nucleus). Within the hotspots, the relative DACH to platinum ratio is similar to the average cellular value in SW480 cells, but drastically lower in HCT116 cells (wt and OxR). The distribution of the oxalate ligand (leaving group) relative to platinum (B) indicates a disproportionally higher accumulation of oxalate relative to the metal in HCT116 OxR cells. HCT116 and HCT116 OxR cells were exposed to 100 μM oxaliplatin, SW480 cells were exposed to 200 μM for 24 h. Abbreviations: cell – entire cell, cyt – cytoplasm; nu – nucleus; chr – chromatin; mit – mitochondria; hs – hotspots. The slopes of the dashed lines refer to the mean relative ligand to platinum ratio determined on the resin sections of each cell line. Further details about the displayed quantities and their inference from NanoSIMS measurement data are contained in a previous publication^14^ and provided in the materials and method section of this paper, the NanoSIMS data acquired in this study are presented in Fig. S6.† Displayed data points and error bars refer to means ± SD.

The dual stable isotope labeling strategy allowed us to determine the ligand-specific subcellular distribution patterns simultaneously. Both, ^13^C from the (expectedly leaving) oxalate and ^2^H from the (expectedly non-leaving) DACH ligands were shown to accumulate within the treated cells (Fig. 7A, B, S6A and B†). This suggests that a portion of the drug enters the cells without prior ligand cleavage, *i.e.* with both ligands arriving inside the cellular matrix. According to the coordination properties of DACH (non-leaving group) and from the relative linearity of the deuterium and platinum distribution, it can be inferred that in all three cell lines the DACH ligand is still co-localized with and, presumably bound to, the platinum center of the drug, everywhere outside the cytoplasmic platinum hotspots (Fig. 7A and S7A†). On the contrary, the relative DACH to platinum content within the cytoplasmic hotspots is similar to the cell average for SW480 cells, but significantly lower in HCT116 cells, both for the drug sensitive and resistant cell line (Fig. 7A). Similar to DACH, the subcellular distribution pattern of oxalate within each cell line appears rather uniform, revealing no specific compartments with enhanced oxalate accumulation. Strikingly, in HCT116 OxR cells a disproportionally high amount of oxalate relative to platinum was detected, suggesting the selective accumulation of the oxalate ligand in the resistant cells (Fig. 7B and S7B†).

In HCT116 cells (wt and OxR), cytoplasmic hotspots with a high tendency for platinum accumulation (Fig. 2B) were shown to accumulate ^2^H from the labeled DACH ligand and ^13^C from the labeled oxalate ligand to a lower extent than platinum (Fig. 7 and S7†). These data suggest that oxaliplatin, upon entering the specific cytoplasmic organelles (likely of endo-/lysosomal origin), tends to readily lose the labile oxalate and some of the more stably bound DACH ligand. Theoretically, such a scenario is possible if the ligands are exchanged with thiol containing molecules, however, only the SW480 cytoplasmic platinum aggregates were profoundly rich in sulfur (Fig. S2†). The possibility of DACH cleavage from the platinum center of oxaliplatin was confirmed in the presence of specific di-sulfur species *via* MALDI by other authors.^36^

Though the levels of ^2^H and ^13^C isotope enrichment were sufficiently high for label detection within the cell organelles, differential elution and dilution of the drug components as a consequence of resin embedding, which is essential for preservation of the cell ultrastructure and high resolution TEM imaging, may also affect the observed stoichiometry of the drug. Our cryo-based sample preparation route has been tailored for minimizing wash-out of the analyte; however, a precise determination of the drug stoichiometry necessitates further investigation.

## Discussion

The identification of the subcellular targets of chemotherapeutic agents is a key for the improvement of cancer therapy regimens in the future. Recently, novel correlative bioanalytical approaches have evolved and opened new ways of studying the fate of chemotherapeutics on the subcellular level, not only for elucidating their pharmacological targets, but also for the identification of the cellular loci which might be responsible for systemic toxicity and resistance. Oxaliplatin has been applied in the clinics worldwide for more than two decades now and is synergistically effective in combination with 5-fluorouracil and folinic acid (leucovorin) for treatment of colorectal cancers.^37–39^ Yet, there are very little data available concerning its subcellular localization. Here we have investigated the subcellular distribution of ^2^H- and ^13^C-labeled oxaliplatin in three colon cancer cell lines with different drug sensitivity.

Trace element analysis necessitates application of appropriate sample preparation techniques that need to be adjusted for specific experimental requirements. For combining cell ultrastructure investigation by means of TEM with elemental and isotopic imaging by NanoSIMS, cryo-based sample preparation was successfully employed on cell monolayers. High-pressure freezing in combination with freeze substitution (FS) is a procedure widely accepted as an improvement over conventional chemical fixation and processing at ambient temperature and it has been applied for preparation of cell monolayers grown on sapphire discs and Aclar.^40,41^ With the introduction of a rapid FS approach, the sample preparation can be significantly accelerated (1/2 day instead of 3–5 days), thereby further reducing wash-out effects.^28^ In cryo-prepared SW480 cell monolayers, cisplatin showed a pronounced aggregation in cytoplasmic sulfur-rich organelles, presumably of lysosomal origin, and in nuclear structures (both in nucleoli and chromatin), suggesting no significant redistribution in comparison to conventionally embedded samples,^14^ which suggests a tight bonding of platinum to its cellular targets. The subcellular cisplatin distribution pattern (Fig. S3†) is in agreement with observations in different tumor (HeLa, A2780) and non-malignant cells reported by other authors.^13,42–44^ In these reports, data obtained by TEM, cell fractionation with consecutive ICP-MS analysis and NanoSIMS likewise identified the nucleus as one of the major sites of cisplatin affinity. Whereas no significant accumulation of platinum was observed after treatment with cisplatin in the cytoplasm of HeLa cells,^43^ the drug was shown to interact with autophagosomes and mitochondria in A2780 ovarian carcinoma cells.^13,42^ In line with our results, platinum co-aggregated with sulfur in the cytoplasm of the cells. These cells, in particular platinum-resistant variants (A2780 cisR), are well known to exhibit high levels of metallothionein and glutathione, which might be responsible for drug trapping and, therefore, resistance.^45,46^ The cytoplasmic aggregation might represent products of glutathione-dependent drug inactivation and therefore invokes further investigations, for instance in murine tissues. It should be taken into account that clinically relevant oxaliplatin concentrations in mice might be below the detection limit of the presented technique and necessitate novel strategies for investigating the corresponding metabolites involved, *e.g.*, the application of Pt(iv) analogues.^23,47,48^

In SW480 cells, the platinum distribution of oxaliplatin is similar to that of cisplatin (Fig. 2B and 4), showing pronounced affinity for nuclei and S-rich cytoplasmic single-membrane bound organelles. Remarkably, nucleoli were barely detectable in sections of SW480 cells exposed to oxaliplatin, whereas they were abundantly present across the entire cell population in the untreated control. Given this observation, fluorescence microscopy was employed and confirmed a pronounced effect of oxaliplatin treatment on nucleolar integrity and distribution (Fig. 3 and S8†). The nucleolus is a major site of pre-ribosomal particle biogenesis and nuclear stress response^49,50^ and has long been recognized as a target for various classes of antineoplastic agents including platinum-based drugs.^51^ In agreement with our data, the nucleolar susceptibility to oxaliplatin was previously shown in cancer cells to play a role in response to the treatment^52^ and in dorsal root ganglia sensory nerve cells to be presumably involved in the induction of the undesirable neurotoxic effect.^53^ Cisplatin and oxaliplatin are capable of interference with the mature ribosomes^54^ as well as with single RNA molecules^55,56^ and can induce ribosome biogenesis stress. It has recently been suggested that the latter mechanism might explain why oxaliplatin has a clinical activity profile distinct from that of cisplatin, which may enable a mechanistically guided selection of front-line cancer chemotherapeutics.^7^ We clearly show that the nucleolar structure in human colon cancer cells is a major site highly impacted by accumulation of both cisplatin and oxaliplatin and deserves closer attention in future search for specific targets of platinum-based drugs. The cytotoxic effect of 5-fluorouracil, broadly used in adjuvant chemotherapy in combination with oxaliplatin, has been demonstrated to involve incorporation into RNA as well as thymidylate synthase inhibition and to be linked to inhibition of nucleolar function,^57,58^ rendering the nucleolus a putative node for the synergistic effect of both drugs. Taken together, the nucleolus – a site of close association of RNA and DNA molecules – might be a target for oxaliplatin and other nuclear targeting platinum-based drug candidates,^15,59^ and possible interactions of platinum compounds with this organelle should be investigated in more detail. In particular, the reported approach paves the way for addressing questions such as whether spatio-temporal co-accumulation of drugs could be responsible for their synergism (*e.g.*, 5-fluorourocil and oxaliplatin).

Increased efflux and decreased influx are widely recognized as resistance mechanisms to platinum-based drugs.^60^ In the isogenic pair of HCT116 wt and HCT116 OxR cells, the difference in cellular accumulation of oxaliplatin (Table 1, 30% higher accumulation in sensitive HCT116 wt cells) is clearly insufficient to explain the high degree of insensitivity of the latter (resistance factor >20-fold). In order to investigate whether the subcellular localization plays a specific role in drug resistance, we compared the platinum distribution in both cell lines. In accordance with ICP-MS data, the SIMS analysis confirmed the little higher accumulation of the drug in HCT116 wt cells in comparison to HCT116 OxR cells. Equivalent to SW480 cells, oxaliplatin was prone to accumulation in cytoplasmic single-membrane bound vesicles and nuclear structures in HCT116 cell lines (Fig. 2 and 5). In contrast to SW480 cells, the cytoplasmic platinum hotspots were relatively depleted in sulfur and nitrogen (Fig. S5†) and the platinum levels in mitochondria were significantly higher than the average cytoplasmic values (Fig. 5), suggesting that possible targets of the drug may include the mitochondria in HCT116 cells. The latter is in good agreement with the reported mitochondrial apoptotic response in enucleated HCT116 cells, which can be initiated by interaction of oxaliplatin with cellular structures other than nuclear DNA.^61^

The dual stable isotope labeling strategy has successfully been employed by other authors for simultaneous determination of the distribution of the metal and the ligands after cancer cell exposure to ruthenium(ii)-based RAPTA-C complex.^12^ By application of the NanoSIMS-based approach, the divergent distribution of drug components was revealed, as the labile ^13^C-labeled arene group of RAPTA-C showed rather homogenous distribution, in contrast to ^15^N-labeled phosphatricyclodecane that tended to colocalize with ruthenium in close association with plasma membrane proteins or the close extracellular matrix. Here, we report the non-leaving ^2^H-labeled DACH ligand to co-accumulate with platinum within subcellular structures (including nuclear structures and cytoplasm) of oxaliplatin treated SW480, HCT116 and HCT116 OxR cells (Fig. 7A), with the only exception of cytoplasmic hotspots. The ^13^C-labeled oxalate – the leaving group of oxaliplatin – was shown to distribute rather uniformly within the cells. Interestingly, in HCT116 OxR cells the expected amount of oxalate, when related to the platinum content, should not exceed the amount of oxalate in the parental HCT116 cells; however, it comes close to the levels observed in SW480 cells treated with a twice higher concentration and a more than 3-fold higher drug accumulation in general (Fig. 7B, Table 1). The resistant cells have a tendency for oxalate sequestration unparalleled to DACH and platinum accumulation. This observation raises the intriguing question whether oxalate accumulation *per se* might be involved in the development of the acquired resistance.

Oxalate – a typical plant cell resident molecule – is regarded as a terminal product of ascorbic acid metabolism in animal cells and forms salts with abundant divalent metallic cations (*e.g.* Mg^2+^, Fe^2+^, Ca^2+^).^62,63^ In the presence of Mg^2+^ and Ca^2+^ ions the decomposition rates of oxaliplatin in solution can be significantly increased.^64^ The co-incubation of oxaliplatin with CaCl_2_ results in formation of the biologically active dichlorido platinum complex and calcium oxalate crystals, shifting the reaction equilibrium to the side of the products.^65^ In the human body, calcium chelation by the oxalate ligand is regarded as a possible reason for the most profound adverse side effect of oxaliplatin – peripheral neuropathy. As a consequence, parallel calcium and magnesium infusions were recommended to reduce the oxaliplatin-induced neurotoxicity.^4,66^ Finally, calcium oxalate is known to contribute to kidney injury through induction of an epithelial-to-mesenchymal transition in the proximal tubular epithelial cells.^67^ With respect to the data of oxalate interactions *in vitro* and *in vivo*, several scenarios about its potential role in the oxaliplatin resistant HCT116 cell line are conceivable: (I) the subcellular accumulation of oxalate salts with divalent cations inhibits the passive diffusion of the platinum salt through the cell membranes; (II) Ca^2+^ depletion inside the cell results in a decreased oxaliplatin conversion into the active dichlorido platinum species; (III) chelation of Ca^2+^ ions depletes the secondary messenger pool required for apoptosis induction,^68^ rendering the cells less responsive to drug-induced damage.

Overall, the multiple isotope labeling strategy combined with trace element and isotope analysis by NanoSIMS emerges as a powerful tool for exploring the distribution of clinically relevant drugs on the sub-cellular scale. As demonstrated by our study on oxaliplatin, this experimental/analytical approach provides the ability to study the distribution of the metal centers in parallel to those of the metal-bound/released ligands.

## Conclusion

Oxaliplatin is known to form different types of DNA lesions including crosslinks that are potentially lethal to cells, but, recently, interaction with rRNA and ribosomal biogenesis stress have been emphasized as key factors for its cytotoxicity. Our findings in human colon cancer cell lines confirm a pronounced accumulation of oxaliplatin (and cisplatin) in the nucleolus – a major site of RNA/DNA interaction and ribosome biogenesis – and support the hypothesis that it may be a critical target for platinum-based chemotherapy. *Via* application of the dual stable isotope labeling strategy, a striking difference between oxaliplatin sensitive and resistant HCT116 cells was revealed: a pronounced oxalate accumulation in the latter. Whether the oxalate accumulation effects the acquired resistance on the cellular and/or organismal level (in patients) deserves further investigation. Broader application of chemical imaging techniques suitable for studying the subcellular localization of clinically relevant agents bearing isotopic labels opens new horizons in the field of chemical pharmacology.

## Conflicts of interest

The authors declare no potential conflicts of interest.

## Abbreviations

AFS: Automated freeze substitution
DACH: Diaminocyclohexane
EM: Electron microscopy
FITC: Fluorescein isothiocyanate
FS: Freeze substitution
ICP-MS: Inductively coupled plasma mass spectrometry
MALDI: Matrix-assisted laser desorption/ionization
NanoSIMS: Nanometer-scale secondary ion mass spectrometry
QSA: Quasi-simultaneous arrival
ROI: Region(s) of interest
TEM: Transmission electron microscopy

## Supplementary Material

NA-003-D0NA00685H-s001

## References

[cit1] Raymond E., Faivre S., Chaney S., Woynarowski J., Cvitkovic E. (2002). Cellular and molecular pharmacology of oxaliplatin. Mol. Cancer Ther..

[cit2] Andre T., Boni C., Navarro M., Tabernero J., Hickish T., Topham C., Bonetti A., Clingan P., Bridgewater J., Rivera F., de Gramont A. (2009). Improved overall survival with oxaliplatin, fluorouracil, and leucovorin as adjuvant treatment in stage II or III colon cancer in the MOSAIC trial. J. Clin. Oncol..

[cit3] Bokemeyer C., Bondarenko I., Makhson A., Hartmann J. T., Aparicio J., de Braud F., Donea S., Ludwig H., Schuch G., Stroh C., Loos A. H., Zubel A., Koralewski P. (2009). Fluorouracil, leucovorin, and oxaliplatin with and without cetuximab in the first-line treatment of metastatic colorectal cancer. J. Clin. Oncol..

[cit4] Saif M. W., Reardon J. (2005). Management of oxaliplatin-induced peripheral neuropathy. Ther. Clin. Risk Manage..

[cit5] Woynarowski J. M., Chapman W. G., Napier C., Herzig M. C., Juniewicz P. (1998). Sequence- and region-specificity of oxaliplatin adducts in naked and cellular DNA. Mol. Pharmacol..

[cit6] Kim Y.-S., Shin S.-M., Cheong M.-S., Hah S.-S. (2010). Mechanistic Insights into in vitro DNA Adduction of Oxaliplatin. Bull. Korean Chem. Soc..

[cit7] Bruno P. M., Liu Y., Park G. Y., Murai J., Koch C. E., Eisen T. J., Pritchard J. R., Pommier Y., Lippard S. J., Hemann M. T. (2017). A subset of platinum-containing chemotherapeutic agents kills cells by inducing ribosome biogenesis stress. Nature Medicine.

[cit8] Ozdian T., Holub D., Maceckova Z., Varanasi L., Rylova G., Rehulka J., Vaclavkova J., Slavik H., Moudry P., Znojek P., Stankova J., de Sanctis J. B., Hajduch M., Dzubak P. (2017). Proteomic profiling reveals DNA damage, nucleolar and ribosomal stress are the main responses to oxaliplatin treatment in cancer cells. J. Proteomics.

[cit9] Lee R. F. S., Theiner S., Meibom A., Koellensperger G., Keppler B. K., Dyson P. J. (2017). Application of imaging mass spectrometry approaches to facilitate metal-based anticancer drug research. Metallomics.

[cit10] Chandra S. (2010). Quantitative imaging of chemical composition in single cells by secondary ion mass spectrometry: cisplatin affects calcium stores in renal epithelial cells. Methods Mol. Biol..

[cit11] Proetto M. T., Callmann C. E., Cliff J., Szymanski C. J., Hu D., Howell S. B., Evans J. E., Orr G., Gianneschi N. C. (2018). Tumor Retention of Enzyme-Responsive Pt(II) Drug-Loaded Nanoparticles Imaged by Nanoscale Secondary Ion Mass Spectrometry and Fluorescence Microscopy. ACS Cent. Sci..

[cit12] Lee R. F. S., Escrig S., Croisier M., Clerc-Rosset S., Knott G. W., Meibom A., Davey C. A., Johnsson K., Dyson P. J. (2015). NanoSIMS analysis of an isotopically labelled organometallic ruthenium(II) drug to probe its distribution and state in vitro. Chem. Commun..

[cit13] Lee R. F. S., Riedel T., Escrig S., Maclachlan C., Knott G. W., Davey C. A., Johnsson K., Meibom A., Dyson P. J. (2017). Differences in cisplatin distribution in sensitive and resistant ovarian cancer cells: a TEM/NanoSIMS study. Metallomics.

[cit14] Legin A. A., Schintlmeister A., Jakupec M. A., Galanski M., Lichtscheidl I., Wagner M., Keppler B. K. (2014). NanoSIMS combined with fluorescence microscopy as a tool for subcellular imaging of isotopically labeled platinum-based anticancer drugs. Chem. Sci..

[cit15] Wedlock L. E., Kilburn M. R., Liu R., Shaw J. A., Berners-Price S. J., Farrell N. P. (2013). NanoSIMS multi-element imaging reveals internalisation and nucleolar targeting for a highly-charged polynuclear platinum compound. Chem. Commun..

[cit16] Wedlock L. E., Kilburn M. R., Cliff J. B., Filgueira L., Saunders M., Berners-Price S. J. (2011). Visualising gold inside tumour cells following treatment with an antitumour gold(I) complex. Metallomics.

[cit17] Lechene C., Hillion F., McMahon G., Benson D., Kleinfeld A. M., Kampf J. P., Distel D., Luyten Y., Bonventre J., Hentschel D., Park K. M., Ito S., Schwartz M., Benichou G., Slodzian G. (2006). High-resolution quantitative imaging of mammalian and bacterial cells using stable isotope mass spectrometry. Journal of Biology.

[cit18] Carpenter K. J., Weber P. K., Davisson M. L., Pett-Ridge J., Haverty M. I., Keeling P. J. (2013). Correlated SEM, FIB-SEM, TEM, and NanoSIMS imaging of microbes from the hindgut of a lower termite: methods for in situ functional and ecological studies of uncultivable microbes. Microsc. 1Microanal..

[cit19] Finzi-Hart J. A., Pett-Ridge J., Weber P. K., Popa R., Fallon S. J., Gunderson T., Hutcheon I. D., Nealson K. H., Capone D. G. (2009). Fixation and fate of C and N in the cyanobacterium *Trichodesmium* using nanometer-scale secondary ion mass spectrometry. Proc. Natl. Acad. Sci. U. S. A..

[cit20] Chandra S. (2008). Subcellular imaging of RNA distribution and DNA replication in single mammalian cells with SIMS: the localization of heat shock induced RNA in relation to the distribution of intranuclear bound calcium. Journal of Microscopy.

[cit21] Georgantzopoulou A., Serchi T., Cambier S., Leclercq C. C., Renaut J., Shao J., Kruszewski M., Lentzen E., Grysan P., Eswara S., Audinot J. N., Contal S., Ziebel J., Guignard C., Hoffmann L., Murk A. J., Gutleb A. C. (2016). Effects of silver nanoparticles and ions on a co-culture model for the gastrointestinal epithelium. Part. Fibre Toxicol..

[cit22] He C., Fong L. G., Young S. G., Jiang H. (2017). NanoSIMS imaging: an approach for visualizing and quantifying lipids in cells and tissues. J. Invest. Med..

[cit23] Legin A. A., Theiner S., Schintlmeister A., Reipert S., Heffeter P., Jakupec M. A., Mayr J., Varbanov H. P., Kowol C. R., Galanski M., Berger W., Wagner M., Keppler B. K. (2016). Multi-scale imaging of anticancer platinum(iv) compounds in murine tumor and kidney. Chem. Sci..

[cit24] Wedlock L. E., Berners-Price S. J. (2011). Recent advances in mapping the sub-cellular distribution of metal-based anticancer drugs. Aust. J. Chem..

[cit25] Habala L., Galanski M., Yasemi A., Nazarov A. A., von Keyserlingk N. G., Keppler B. K. (2005). Synthesis and structure-activity relationships of mono- and dialkyl-substituted oxaliplatin derivatives. Eur. J. Med. Chem..

[cit26] Slodzian G., Hillion F., Stadermann F. J., Zinner E. (2004). QSA influences on isotopic ratio measurements. Appl. Surf. Sci..

[cit27] Jimenez N., Humbel B. M., van Donselaar E., Verkleij A. J., Burger K. N. (2006). Aclar discs: a versatile substrate for routine high-pressure freezing of mammalian cell monolayers. Journal of Microscopy.

[cit28] Goldammer H., Hollergschwandtner E., Elisabeth N. H., Frade P. R., Reipert S. (2016). Automatized Freeze Substitution of Algae Accelerated by a Novel Agitation Module. Protist.

[cit29] Reipert S., Goldammer H., Richardson C., Goldberg M. W., Hawkins T. J., Hollergschwandtner E., Kaufmann W. A., Antreich S., Stierhof Y. D. (2018). Agitation Modules: Flexible Means to Accelerate Automated Freeze Substitution. J. Histochem. Cytochem..

[cit30] Seetharam R., Sood A., Goel S. (2009). Oxaliplatin: pre-clinical perspectives on the mechanisms of action, response and resistance. Ecancermedicalscience.

[cit31] Wang D., Lippard S. J. (2005). Cellular processing of platinum anticancer drugs. Nat. Rev. Drug Discovery.

[cit32] Chaney S. G., Campbell S. L., Bassett E., Wu Y. (2005). Recognition and processing of cisplatin- and oxaliplatin-DNA adducts. Critical Reviews in Oncology/Hematology.

[cit33] Reynolds R. C., Montgomery P. O., Hughes B. (1964). Nucleolar "Caps" Produced by Actinomycin D. Cancer Research.

[cit34] Shav-Tal Y., Blechman J., Darzacq X., Montagna C., Dye B. T., Patton J. G., Singer R. H., Zipori D. (2005). Dynamic sorting of nuclear components into distinct nucleolar caps during transcriptional inhibition. Mol. Biol. Cell.

[cit35] Safaei R., Larson B. J., Cheng T. C., Gibson M. A., Otani S., Naerdemann W., Howell S. B. (2005). Abnormal lysosomal trafficking and enhanced exosomal export of cisplatin in drug-resistant human ovarian carcinoma cells. Mol. Cancer Ther..

[cit36] Liu X., Hummon A. B. (2016). Chemical Imaging of Platinum-Based Drugs and their Metabolites. Sci. Rep..

[cit37] Raymond E., Chaney S. G., Taamma A., Cvitkovic E. (1998). Oxaliplatin: A review of preclinical and clinical studies. Annals of Oncology.

[cit38] Conroy T., Desseigne F., Ychou M., Bouche O., Guimbaud R., Becouarn Y., Adenis A., Raoul J. L., Gourgou-Bourgade S., de la Fouchardiere C., Bennouna J., Bachet J. B., Khemissa-Akouz F., Pere-Verge D., Delbaldo C., Assenat E., Chauffert B., Michel P., Montoto-Grillot C., Ducreux M. (2011). FOLFIRINOX versus gemcitabine for metastatic pancreatic cancer. N. Engl. J. Med..

[cit39] Alcindor T., Beauger N. (2011). Oxaliplatin: a review in the era of molecularly targeted therapy. Curr. Oncol..

[cit40] Reipert S. and Wiche G., High-Pressure Freezing and Low-Temperature Fixation of Cell Monolayers Grown on Sapphire Coverslips, Methods Cell Biol., Elsevier, 2008, vol. 88, pp. 165–180 10.1016/S0091-679X(08)00410-X 18617034

[cit41] Jimenez N., Humbel B. M., van Donselaar E., Verkeleij A. J., Burger K. N. J. (2006). Aclar discs: a versatile substrate for routine high-pressure freezing of mammalian cell monolayers. J. Microsc..

[cit42] Groessl M., Zava O., Dyson P. J. (2011). Cellular uptake and subcellular distribution of ruthenium-based metallodrugs under clinical investigation versus cisplatin. Metallomics.

[cit43] Khan M. U., Sadler P. J. (1978). Distribution of a platinum anti-tumour drug in HeLa cells by analytical electron microscopy. Chem.-Biol. Interact..

[cit44] Usami N., Furusawa Y., Kobayashi K., Lacombe S., Reynaud-Angelin A., Sage E., Wu T. D., Croisy A., Guerquin-Kern J. L., Le Sech C. (2008). Mammalian cells loaded with platinum-containing molecules are sensitized to fast atomic ions. Int. J. Radiat. Biol..

[cit45] Okuno S., Sato H., Kuriyama-Matsumura K., Tamba M., Wang H., Sohda S., Hamada H., Yoshikawa H., Kondo T., Bannai S. (2003). Role of cystine transport in intracellular glutathione level and cisplatin resistance in human ovarian cancer cell lines. Br. J. Cancer.

[cit46] Surowiak P., Materna V., Maciejczyk A., Pudelko M., Markwitz E., Spaczynski M., Dietel M., Zabel M., Lage H. (2007). Nuclear metallothionein expression correlates with cisplatin resistance of ovarian cancer cells and poor clinical outcome. Virchows Arch..

[cit47] Lambert I. H., Sorensen B. H. (2018). Facilitating the Cellular Accumulation of Pt-Based Chemotherapeutic Drugs. Int. J. Mol. Sci..

[cit48] Johnstone T. C., Suntharalingam K., Lippard S. J. (2016). The Next Generation of Platinum Drugs: Targeted Pt(II) Agents, Nanoparticle Delivery, and Pt(IV) Prodrugs. Chem. Rev..

[cit49] Shaw P. J., Jordan E. G. (1995). The nucleolus. Annu. Rev. Cell Dev. Biol..

[cit50] Boulon S., Westman B. J., Hutten S., Boisvert F. M., Lamond A. I. (2010). The nucleolus under stress. Mol. Cell.

[cit51] Pickard A. J., Bierbach U. (2013). The cell's nucleolus: an emerging target for chemotherapeutic intervention. ChemMedChem.

[cit52] Burger K., Muhl B., Harasim T., Rohrmoser M., Malamoussi A., Orban M., Kellner M., Gruber-Eber A., Kremmer E., Holzel M., Eick D. (2010). Chemotherapeutic drugs inhibit ribosome biogenesis at various levels. J. Biol. Chem..

[cit53] McKeage M. J., Hsu T., Screnci D., Haddad G., Baguley B. C. (2001). Nucleolar damage correlates with neurotoxicity induced by different platinum drugs. Br. J. Cancer.

[cit54] Melnikov S. V., Soll D., Steitz T. A., Polikanov Y. S. (2016). Insights into RNA binding by the anticancer drug cisplatin from the crystal structure of cisplatin-modified ribosome. Nucleic Acids Res..

[cit55] Chapman E. G., Hostetter A. A., Osborn M. F., Miller A. L., DeRose V. J. (2011). Binding of kinetically inert metal ions to RNA: the case of platinum(II). Met. Ions Life Sci..

[cit56] Becker J. P., Weiss J., Theile D. (2014). Cisplatin, oxaliplatin, and carboplatin unequally inhibit in vitro mRNA translation. Toxicol. Lett..

[cit57] Noordhuis P., Holwerda U., Van der Wilt C. L., Van Groeningen C. J., Smid K., Meijer S., Pinedo H. M., Peters G. J. (2004). 5-Fluorouracil incorporation into RNA and DNA in relation to thymidylate synthase inhibition of human colorectal cancers. Annals of Oncology.

[cit58] Ghoshal K., Jacob S. T. (1997). An alternative molecular mechanism of action of 5-fluorouracil, a potent anticancer drug. Biochem. Pharmacol..

[cit59] Botchway S. W., Charnley M., Haycock J. W., Parker A. W., Rochester D. L., Weinstein J. A., Williams J. A. (2008). Time-resolved and two-photon emission imaging microscopy of live cells with inert platinum complexes. Proc. Natl. Acad. Sci. U. S. A..

[cit60] Heffeter P., Jungwirth U., Jakupec M., Hartinger C., Galanski M., Elbling L., Micksche M., Keppler B., Berger W. (2008). Resistance against novel anticancer metal compounds: differences and similarities. Drug Resist. Updates.

[cit61] Gourdier I., Crabbe L., Andreau K., Pau B., Kroemer G. (2004). Oxaliplatin-induced mitochondrial apoptotic response of colon carcinoma cells does not require nuclear DNA. Oncogene.

[cit62] Knight J., Madduma-Liyanage K., Mobley J. A., Assimos D. G., Holmes R. P. (2016). Ascorbic acid intake and oxalate synthesis. Urolithiasis.

[cit63] Franceschi V. R., Nakata P. A. (2005). Calcium oxalate in plants: formation and function. Annu. Rev. Plant Biol..

[cit64] Han C. H., Khwaounjoo P., Hill A. G., Miskelly G. M., McKeage M. J. (2017). Predicting effects on oxaliplatin clearance: in vitro, kinetic and clinical studies of calcium- and magnesium-mediated oxaliplatin degradation. Sci. Rep..

[cit65] Hizal S., Hejl M., Jungmann C., Jakupec M. A., Galanski M., Keppler B. K. (2019). Synthesis, Characterization, Cytotoxicity, and Time-Dependent NMR Spectroscopic Studies of (SP-4-3)-Oxalato[(1R,2R,4R/1S,2S,4S)-(4-trifluoromethyl-cyclohexane-1,2-diamine)]platinum(II). Eur. J. Inorg. Chem..

[cit66] Gamelin L., Boisdron-Celle M., Delva R., Guerin-Meyer V., Ifrah N., Morel A., Gamelin E. (2004). Prevention of oxaliplatin-related neurotoxicity by calcium and magnesium infusions: A retrospective study of 161 patients receiving oxaliplatin combined with 5-fluorouracil and leucovorin for advanced colorectal cancer. Clin. Cancer Res..

[cit67] Convento M. B., Pessoa E. A., Cruz E., da Gloria M. A., Schor N., Borges F. T. (2017). Calcium oxalate crystals and oxalate induce an epithelial-to-mesenchymal transition in the proximal tubular epithelial cells: Contribution to oxalate kidney injury. Sci. Rep..

[cit68] Pinton P., Giorgi C., Siviero R., Zecchini E., Rizzuto R. (2008). Calcium and apoptosis: ER-mitochondria Ca2+ transfer in the control of apoptosis. Oncogene.

