# Supplementary material for: Nano-scale imaging of dual stable isotope labeled oxaliplatin in human colon cancer cells reveals the nucleolus as a putative node for therapeutic effect
Source: Nanoscale Adv. 2020 Nov 26;3(1):249–62. doi: 10.1039/d0na00685h (PMC9419577; doi:10.1039/d0na00685h)
Supplement: NA-003-D0NA00685H-s001 [file NA-003-D0NA00685H-s001.pdf]

Electronic Supplementary Material (ESI) for Nanoscale Advances.
This journal is © The Royal Society of Chemistry 2022

Supporting information

Nano-scale imaging of dual stable isotope labeled oxaliplatin in human colon cancer cells reveals the nucleolus as a putative node for therapeutic effect

Anton A. Legin[z,y,q*], Arno Schintlmeister[x,q], Nadine S. Sommerfeld[z], Margret Eckhard[w], Sarah Theiner[z,y], Siegfried Reipert[w], Daniel Strohhofer[z], Michael A. Jakupec[z,y,q], Mathea S. Galanski[z], Michael Wagner[x,q] and Bernhard K. Keppler[z,y,q]

[z] Institute of Inorganic Chemistry, Faculty of Chemistry, University of Vienna, A-1090 Vienna, Austria

[y] Research Cluster "Translational Cancer Therapy Research", University of Vienna, A-1090 Vienna, Austria

[q] Research Network "Chemistry meets Microbiology and Environmental Systems Science", University of Vienna, A-1090 Vienna, Austria

[x] Division of Microbial Ecology and Large-Instrument Facility for Environmental and Isotope Research, Centre for Microbiology and Environmental Systems Science, University of Vienna, A-1090 Vienna, Austria

[w] Core Facility of Cell Imaging and Ultrastructure Research, University of Vienna, A-1090 Vienna, Austria

*Corresponding author – Anton A. Legin email: anton.legin@univie.ac.at; T: +43 1 4277 52610; M: +43 650 730 41166; ORCiD ID : 0000-0002-4088-5769

Keywords: oxaliplatin, subcellular distribution, anticancer drugs, stable isotope imaging, correlative microscopy, NanoSIMS

CoI: The authors declare no potential conflicts of interest.

This work was supported by the Austrian Science Fund (FWF) through the project P27749.

Table of contents:

Fig. S1. **Mitochondria and lipid droplets were used to precisely align TEM and SIMS images**. A – electron micrograph of mitochondria containing cristae (arrows) and lipid droplets (asterisks); B – $^{12}C^{14}N^{-}$ secondary ion map indicating the high protein content of mitochondria (bright) in contrast to protein-poor lipid droplets (dark); C – $^{34}S^{-}$ secondary ion map of the same analysis area showing mitochondria as sulfur-rich structures (due to a high iron-sulfur protein content). Note that images A vs. B, C were acquired on two consecutive 75 nm thin sections, respectively. The structural alignment is almost perfect on a low sub-micrometer scale. Intensities are displayed on a false-color scale ranging from low intensities (black) to high intensities (red/white). Scale bar = 1 µm. 4

Fig. S2. **Sulfur-rich platinum aggregates in a SW480 cell treated with 200 µM of oxaliplatin**. Close-up of the cytoplasmic area presented in Fig. 4. Secondary ion maps: A – $^{34}S^{-}$; B – $^{195}Pt^{-}$; C – $^{31}P^{-}$; D – $^{12}C^{14}N^{-}$. Note a weak to no spatial correlation of platinum aggregates with phosphorus and nitrogen. Intensities are displayed on a false-color scale ranging from low intensities (black/blue) to high intensities (white/red). Scale bar = 1 µm. 5

Fig. S3. **Subcellular distribution of cisplatin in SW480 colon cancer cells (24 h, 50 µM)**. A, F – TEM micrographs displaying the cell ultrastructure; B-E – secondary ion intensity distribution images acquired by NanoSIMS. A – cell overview; B – $^{12}C^{14}N^{-}$ secondary ion map referring to the relative elemental nitrogen distribution; C – $^{195}Pt^{-}$ secondary ion map visualizing the drug distribution (hotspots are indicated by the dashed white lines); D – $^{34}S^{-}$ secondary ion map; E – $^{31}P^{-}$ secondary ion map utilized for definition of the nuclear regions (yellow dash). F – high-resolution TEM image of the single-membrane-bound organelles (asterisks) exhibiting strong platinum accumulation (arrowhead in A–E), surrounded by branching mitochondria. Abbreviations: cyt – cytoplasm, nu – nucleus, mit – mitochondria. Intensities are displayed on a false-color scale ranging from low intensities (black/blue) to high intensities (white/red). Scale bars = 5 µm (A-E), 1 µm (F)..6

Fig. S4. **Subcellular platinum distribution in a monolayer of HCT116 *wt* cells after 24 h exposure to 100 µM oxaliplatin**. A, C – TEM micrographs showing the organelles (marked with asterisks) responsible for cytoplasmic platinum accumulation; D, F – overlay of the $^{195}Pt^{-}$ secondary ion map with the corresponding TEM image visualizing the platinum distribution (green) within the cell. B, E – overviews of the corresponding analysis area. Abbreviations: cyt – cytoplasm, mit – mitochondria, nu – nucleus. Scale bar = 5 µm. 7

Fig. S5. **Distribution of selected elements in platinum accumulating structures of HCT116 *wt* and HCT116 OxR cells**. In contrast to platinum-accumulating structures in SW480 cells, morphologically similar organelles in both HCT116 cell lines seem to be depleted both in sulfur and nitrogen. Secondary ion maps: A, E – $^{34}S^{-}$; B, F – $^{195}Pt^{-}$; C, G – $^{12}C^{14}N^{-}$; D, H – $^{31}P^{-}$ acquired on thin sections of 100 µM oxaliplatin treated HCT116 *wt* and HCT116 OxR cells, respectively. Intensities are displayed on a false-color scale ranging from low intensities (black/blue) to high intensities (white/red). Scale bar = 2 µm. 8

Fig. S6. **NanoSIMS measurement data of the local isotopic composition and relative platinum content.** Isotopic compositions are displayed as isotope fractions, given in atom %, as inferred from $^{1}H^{-}$, $^{2}H^{-}$, $^{12}C_2^{-}$ and $^{12}C^{13}C^{-}$ secondary ion signal intensities. HCT116 *wt* and HCT116 OxR cells were exposed to 100 µM of dual-labelled oxaliplatin, SW480 cells were exposed to 200 µM for 24 h. Abbreviations: cell – entire cell, cyt – cytoplasm; ctrl. – untreated SW480 cells, serving as blanks in platinum measurements and natural abundance controls in isotope composition measurements; nu – nucleus; chr – chromatin; mit – mitochondria; hs – hotspots. 9

Fig. S7. **Subcellular distribution of the ligands relative to the metal core with stoichiometry lines (dashed) and variance (dotted)**. This figure supplements the Fig. 7 of the main text, supporting the notion that the distribution of the DACH ligand (non-leaving group) relative to the platinum core (top) shows an almost linear relationship for the major cellular compartments, excluding hotspots, of all 3 cell lines (as the variances for all cell lines are overlapping). The distribution of the oxalate ligand (leaving group) relative to platinum (bottom) indicates a disproportionally higher accumulation of oxalate relative to the metal core in HCT116 OxR cells. The slopes of the dashed lines refer to the mean relative ligand to central atom ratio determined for each cell line. The dotted lines refer to the boundary values

determined by standard deviation of the respective mean. HCT116 and HCT116 OxR cells were exposed to 100 µM oxaliplatin, SW480 cells were exposed to 200 µM for 24 h. Abbreviations: cell – entire cell, cyt – cytoplasm; nuwn – nucleus; chr – chromatin; mit – mitochondria; hs – hotspots. 10

Fig. S8. **Morphological variation of nucleolar structure and number**. Representative fluorescence microscopy images (duplicates) revealing morphological variations in nucleolar structure and abundance within nuclei of SW480 cells upon treatment with actinomycin D (Act D, 1.5 µm) for 4 h and oxaliplatin (OxPt, 200 µM) for 24 h relative to an untreated control. 11

Fig. S9. **Platinum accumulating organelles in HCT116 OxR cells**. TEM micrographs (A, C) showing the cytoplasmic areas of platinum accumulation (asterisks) as judged upon their overlay with NanoSIMS $^{195}Pt^{-}$ secondary ion map (B, D [green pixels]). 12

Fig. S10. **Apoptosis/necrosis induction in SW480 cells after 24 and 48 h exposure to oxaliplatin**, measured by FACS using annexin V-FITC/propidium iodide double-staining. The experiment confirmed the late onset of oxaliplatin toxicity even at 400 µM resulting in less than 20% dead cells after 24 h exposure. The effect is clearly enhancing for the same concentration with the continuation of exposure (>50% dead cells in 48 h). 13

TEM survey images were used for pre-selection of analysis areas subjected to NanoSIMS (performed on consecutive sections). The acquired secondary ion maps were then superimposed on high-resolution electron micrographs utilizing mitochondria and lipid droplets as orientation guides for the spatial correlation (Fig. S1). Mitochondria exhibit characteristic morphological features, which renders them easy to identify in TEM images; in addition, mitochondria show high signal intensities of sulfur and nitrogen associated secondary ion signals which, by combination, allowed us to correlate the ^{195}Pt signal intensity distribution pattern with the cellular ultrastructure.

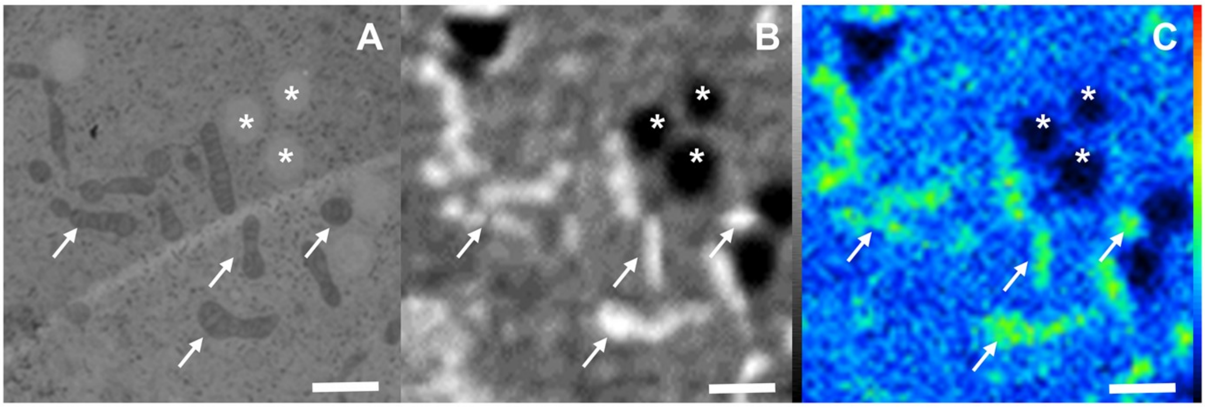

Fig. S1. Mitochondria and lipid droplets were used to precisely align TEM and SIMS images. A – electron micrograph of mitochondria containing cristae (arrows) and lipid droplets (asterisks); B – $^{12}C^{14}N^{-}$ secondary ion map indicating the high protein content of mitochondria (bright) in contrast to protein-poor lipid droplets (dark); C – $^{34}S^{-}$ secondary ion map of the same analysis area showing mitochondria as sulfur-rich structures (due to a high iron-sulfur protein content). Note that images A vs. B, C were acquired on two consecutive 75 nm thin sections, respectively. The structural alignment is almost perfect on a low sub-micrometer scale. Intensities are displayed on a false-color scale ranging from low intensities (black) to high intensities (red/white). Scale bar = 1 µm.

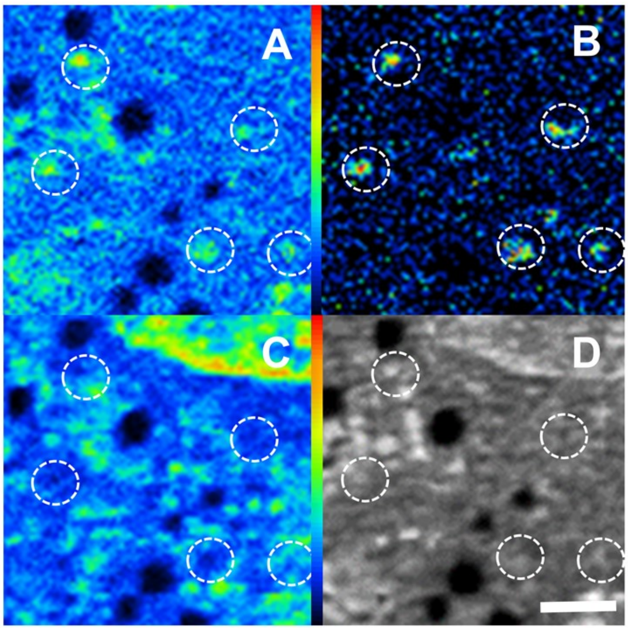

Fig. S2. Sulfur-rich platinum aggregates in a SW480 cell treated with 200 µM of oxaliplatin. Close-up of the cytoplasmic area presented in Fig. 4. Secondary ion maps: A – $^{34}S^{-}$; B – $^{195}Pt^{-}$; C – $^{31}P^{-}$; D – $^{12}C^{14}N^{-}$. Note a weak to no spatial correlation of platinum aggregates with phosphorus and nitrogen. Intensities are displayed on a false-color scale ranging from low intensities (black/blue) to high intensities (white/red). Scale bar = 1 µm.

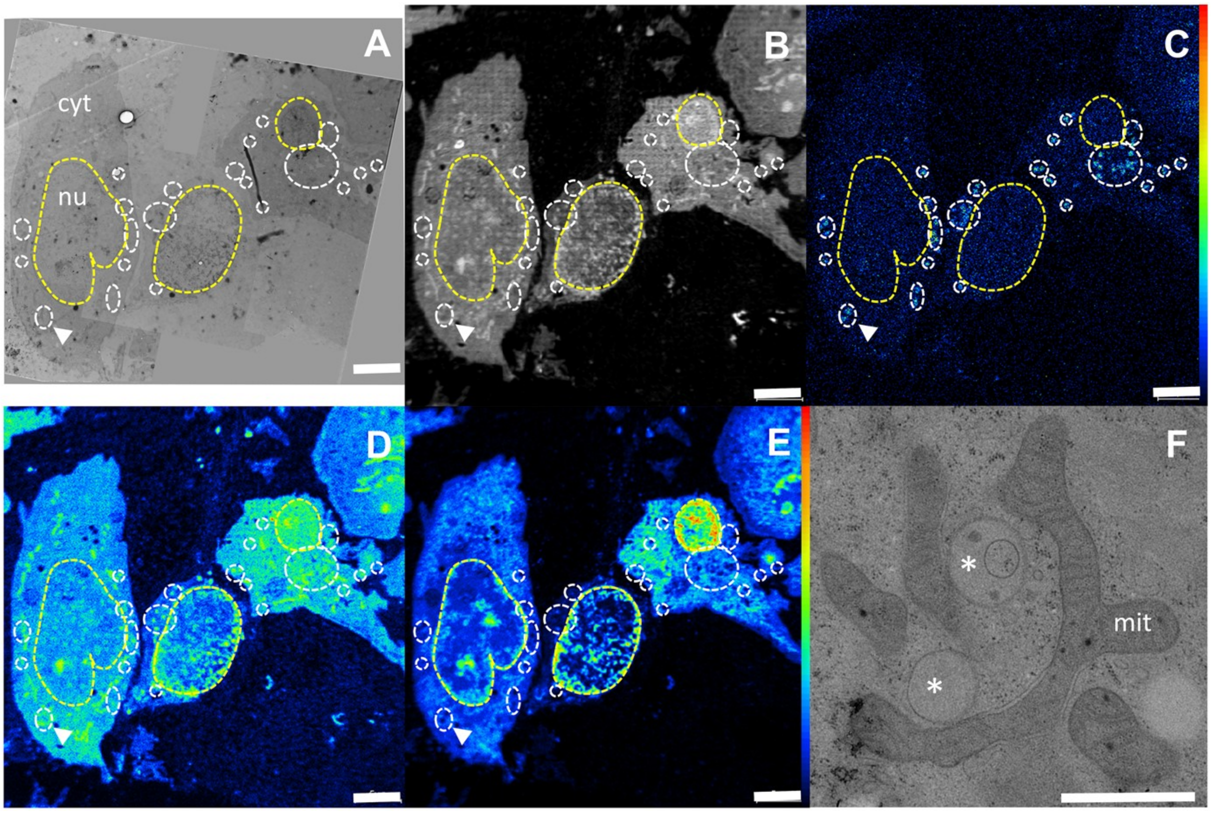

Fig. S3. Subcellular distribution of cisplatin in SW480 colon cancer cells (24 h, 50 µM). A, F – TEM micrographs displaying the cell ultrastructure; B-E – secondary ion intensity distribution images acquired by NanoSIMS. A – cell overview; B – $^{12}C^{14}N^{-}$ secondary ion map referring to the relative elemental nitrogen distribution; C – $^{195}Pt^{-}$ secondary ion map visualizing the drug distribution (hotspots are indicated by the dashed white lines); D – $^{34}S^{-}$ secondary ion map; E – $^{31}P^{-}$ secondary ion map utilized for definition of the nuclear regions (yellow dash). F – high-resolution TEM image of the single-membrane-bound organelles (asterisks) exhibiting strong platinum accumulation (arrowhead in A–E), surrounded by branching mitochondria. Abbreviations: cyt – cytoplasm, nu – nucleus, mit – mitochondria. Intensities are displayed on a false-color scale ranging from low intensities (black/blue) to high intensities (white/red). Scale bars = 5 µm (A-E), 1 µm (F).

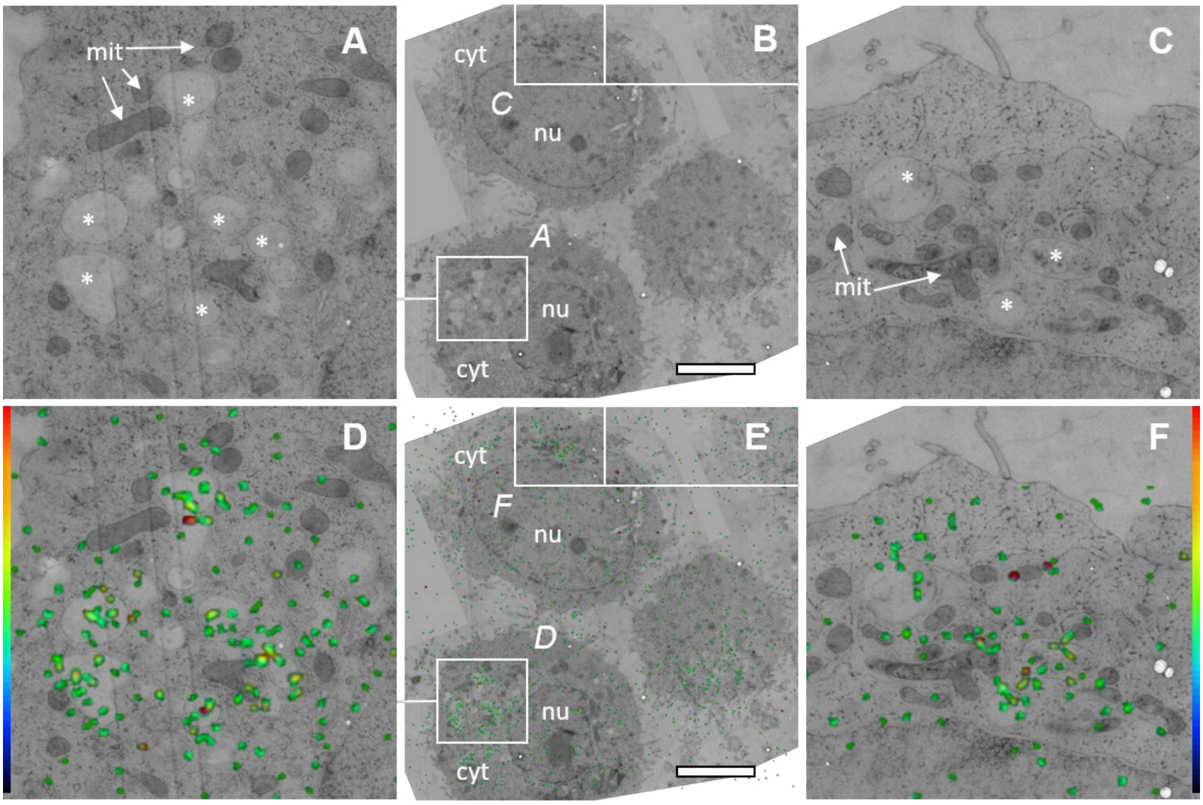

Fig. S4. Subcellular platinum distribution in a monolayer of HCT116 *wt* cells after 24 h exposure to 100 µM oxaliplatin. A, C – TEM micrographs showing the organelles (marked with asterisks) responsible for cytoplasmic platinum accumulation; D, F – overlay of the $^{195}Pt^{-}$ secondary ion map with the corresponding TEM image visualizing the platinum distribution (green) within the cell. B, E – overviews of the corresponding analysis area. Abbreviations: cyt – cytoplasm, mit – mitochondria, nu – nucleus. Intensities are displayed on a false-color scale ranging from low intensities (dark blue) to high intensities (red). Scale bar = 5 µm.

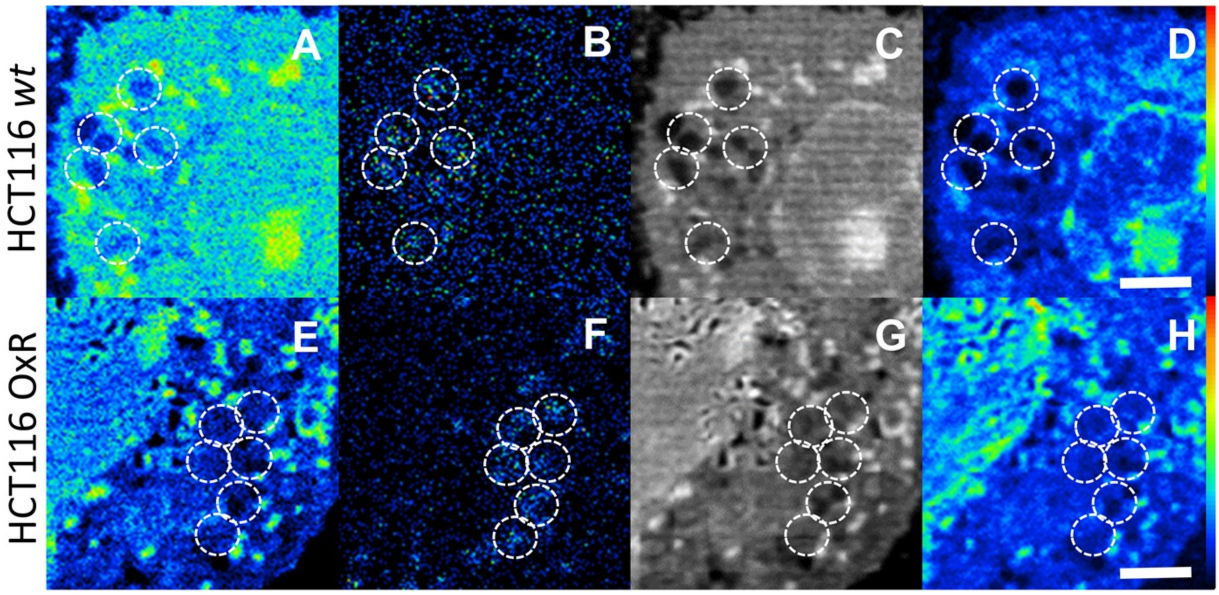

Fig. S5. Distribution of selected elements in platinum accumulating structures of HCT116 *wt* and HCT116 OxR cells. In contrast to platinum-accumulating structures in SW480 cells, morphologically similar organelles in both HCT116 cell lines seem to be depleted both in sulfur and nitrogen. Secondary ion maps: A, E – $^{34}S^{-}$; B, F – $^{195}Pt^{-}$; C, G – $^{12}C^{14}N^{-}$; D, H – $^{31}P^{-}$ acquired on thin sections of 100 µM oxaliplatin treated HCT116 *wt* and HCT116 OxR cells, respectively. Intensities are displayed on a false-color scale ranging from low intensities (black/blue) to high intensities (white/red). Scale bar = 2 µm.

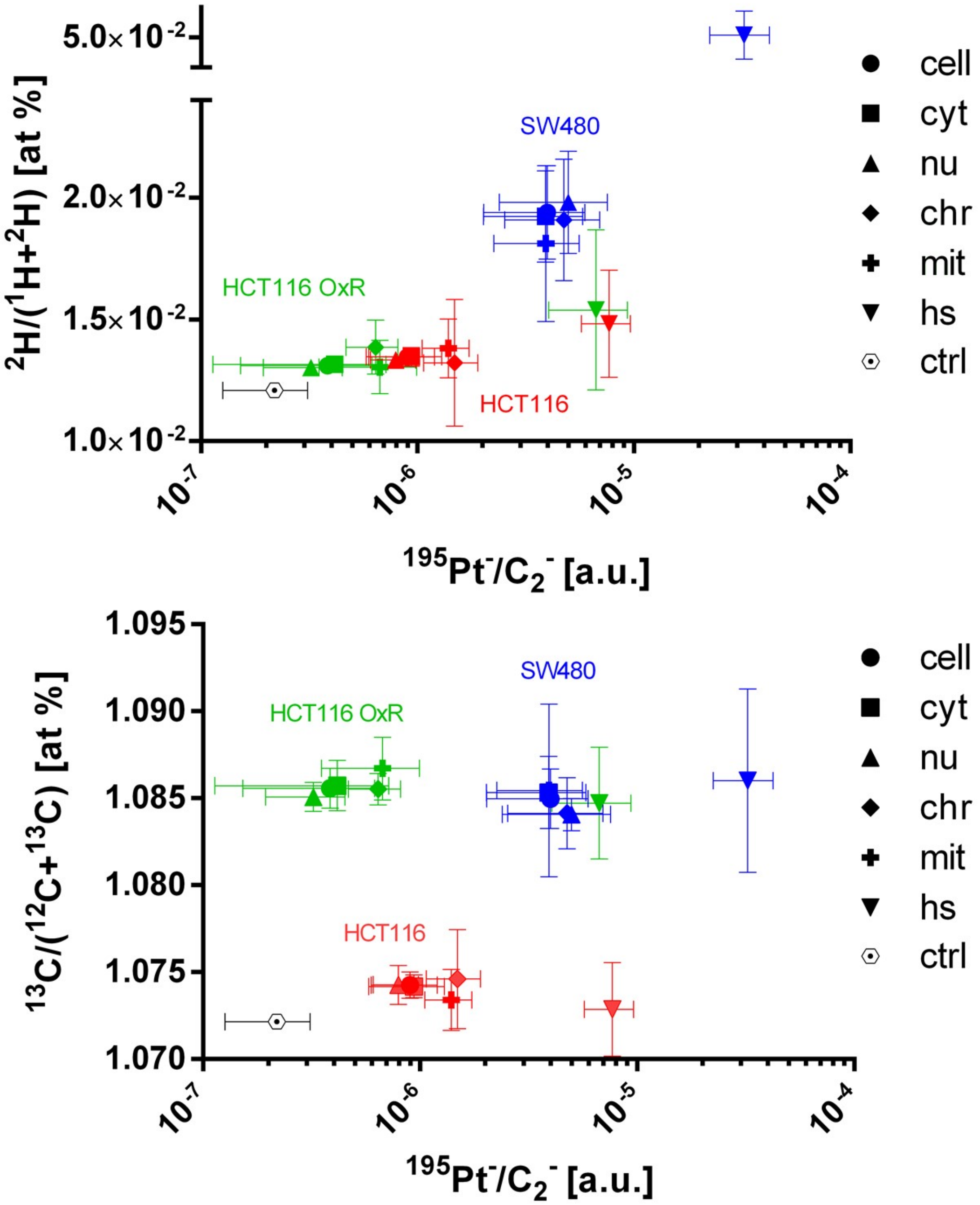

Fig. S6. NanoSIMS measurement data of the local isotopic composition and relative platinum content. Isotopic compositions are displayed as isotope fractions, given in atom %, as inferred from $^{1}H^{-}$, $^{2}H^{-}$, $^{12}C_2^{-}$ and $^{12}C^{13}C^{-}$ secondary ion signal intensities. HCT116 *wt* and HCT116 OxR cells were exposed to 100 µM of dual-labelled oxaliplatin, SW480 cells were exposed to 200 µM for 24 h. Abbreviations: cell – entire cell, cyt – cytoplasm; ctrl – untreated SW480 cells, serving as blanks in platinum measurements and natural abundance controls in isotope composition measurements; nu – nucleus; chr – chromatin; mit – mitochondria; hs – hotspots.

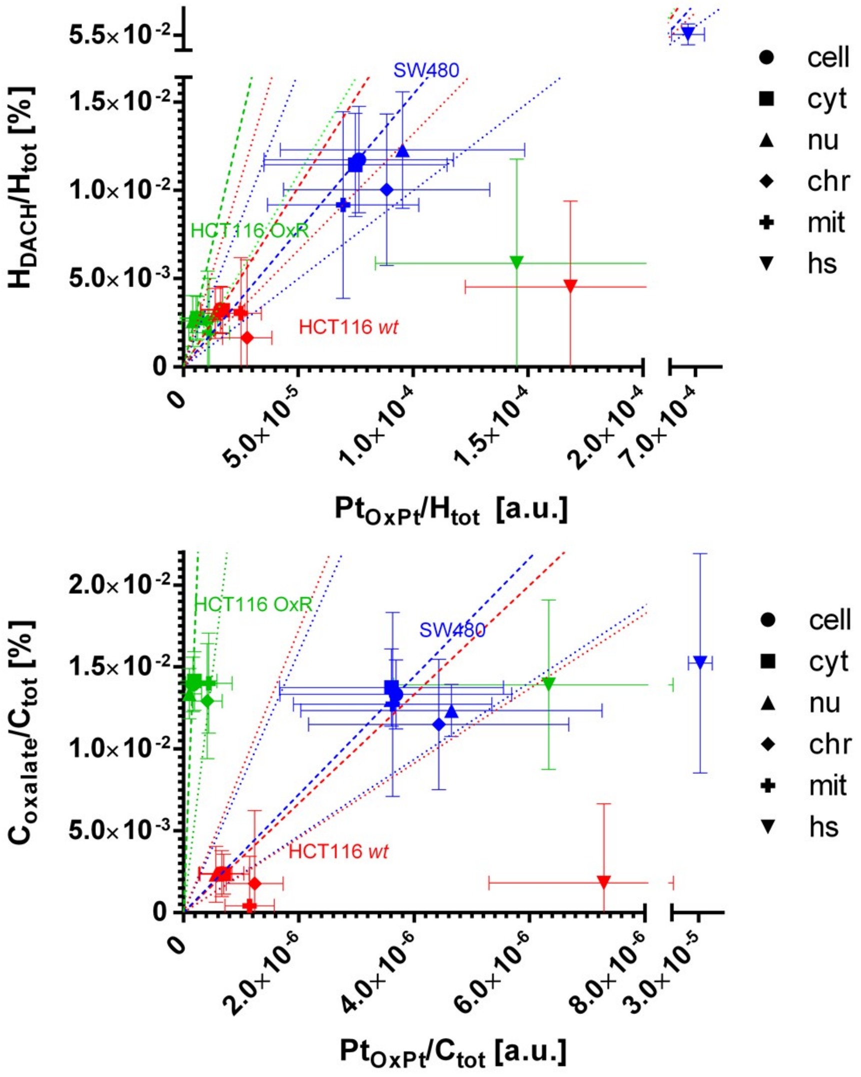

Fig. S7. Subcellular distribution of the ligands relative to the metal core with stoichiometry lines (dashed) and variance (dotted). This figure supplements the Fig. 7 of the main text, supporting the notion that the distribution of the DACH ligand (non-leaving group) relative to the platinum core (top) shows an almost linear relationship for the major cellular compartments, excluding hotspots, of all 3 cell lines (as the variances for all cell lines are overlapping). The distribution of the oxalate ligand (leaving group) relative to platinum (bottom) indicates a disproportionally higher accumulation of oxalate relative to the metal core in HCT116 OxR cells. The slopes of the dashed lines refer to the mean relative ligand to central atom ratio determined for each cell line. The dotted lines refer to the boundary values determined by standard deviation of the respective mean. HCT116 and HCT116 OxR cells were exposed to 100 µM oxaliplatin, SW480 cells were exposed to 200 µM for 24 h. Abbreviations: cell – entire cell, cyt – cytoplasm; nu – nucleus; chr – chromatin; mit – mitochondria; hs – hotspots.

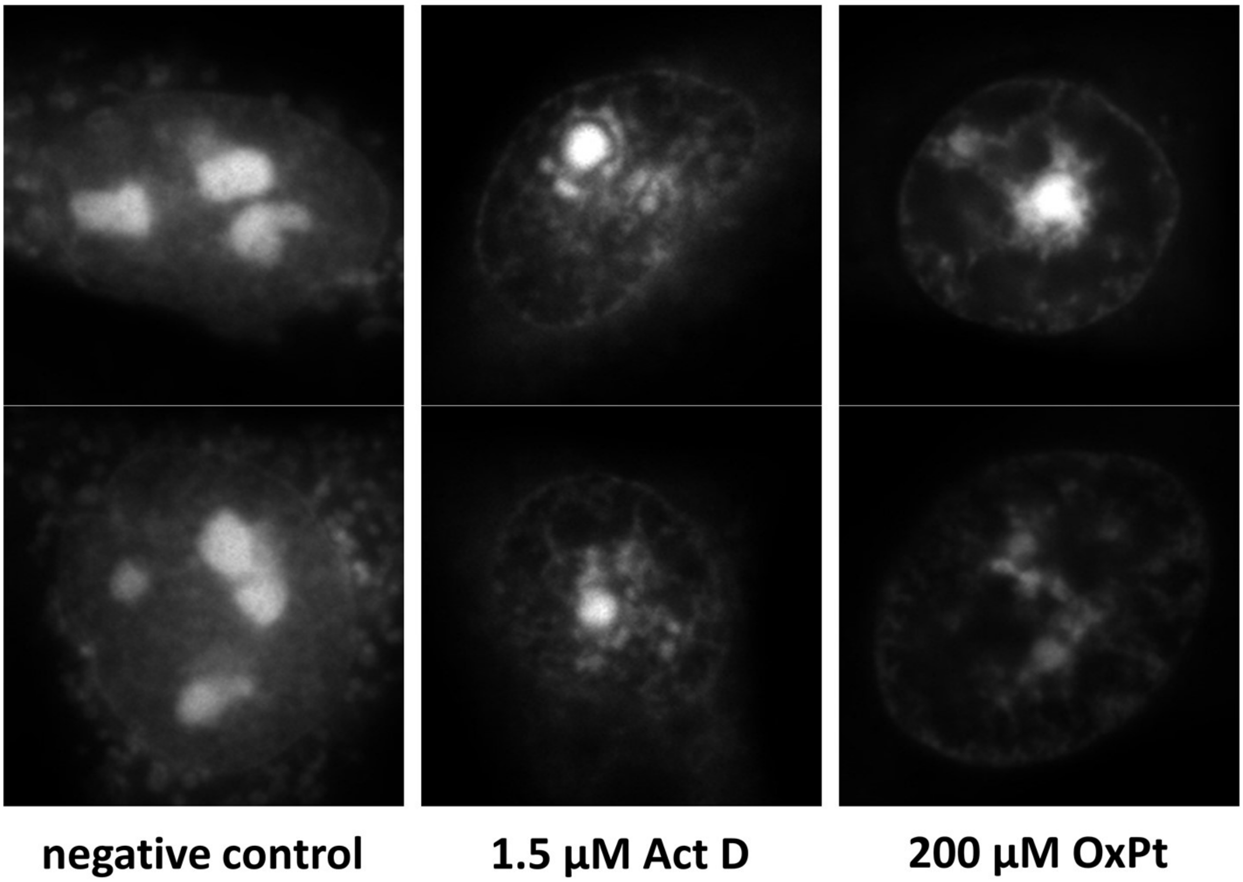

Fig. S8. Morphological variation of nucleolar structure and number. Representative fluorescence microscopy images (duplicates) revealing morphological variations in nucleolar structure and abundance within nuclei of SW480 cells upon treatment with actinomycin D (Act D, 1.5 µm) for 4 h and oxaliplatin (OxPt, 200 µM) for 24 h relative to an untreated control.

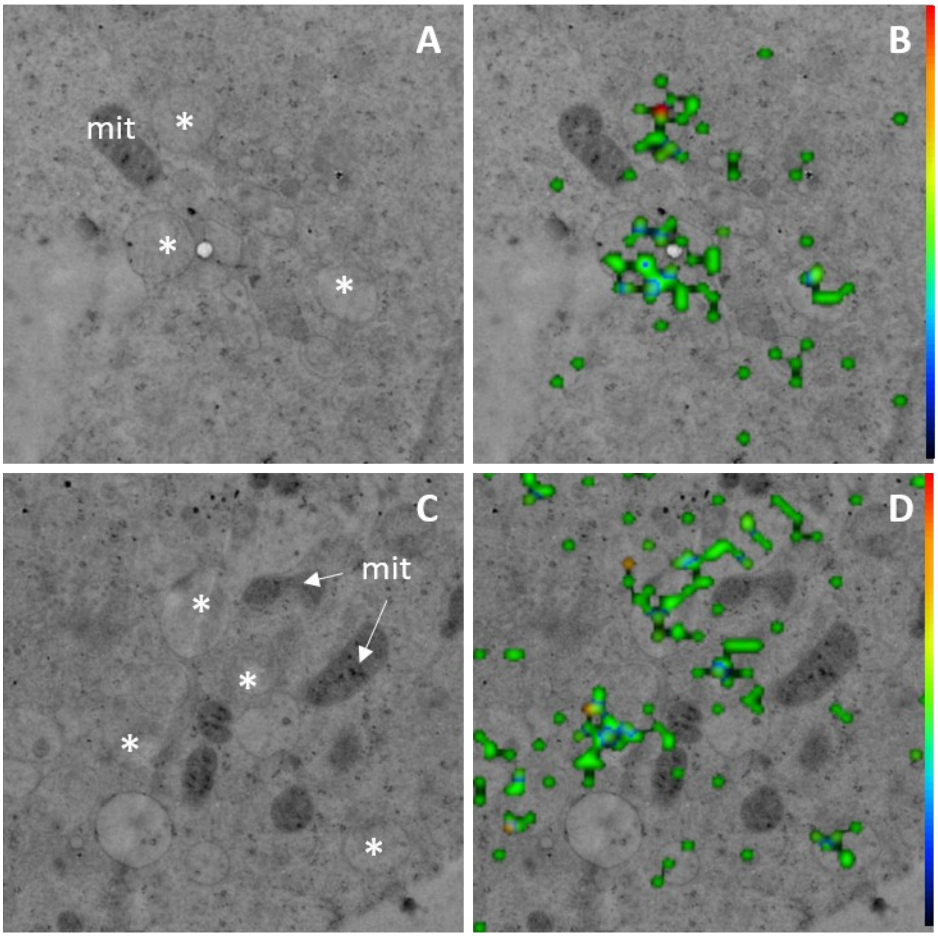

Fig. S9. Platinum accumulating organelles in HCT116 OxR cells. TEM micrographs (A, C) showing the cytoplasmic areas of platinum accumulation (asterisks) as judged upon their overlay with NanoSIMS $^{195}Pt^{-}$ secondary ion map (B, D [colored pixels]). Intensities are displayed on a false-color scale ranging from low intensities (dark blue) to high intensities (red).

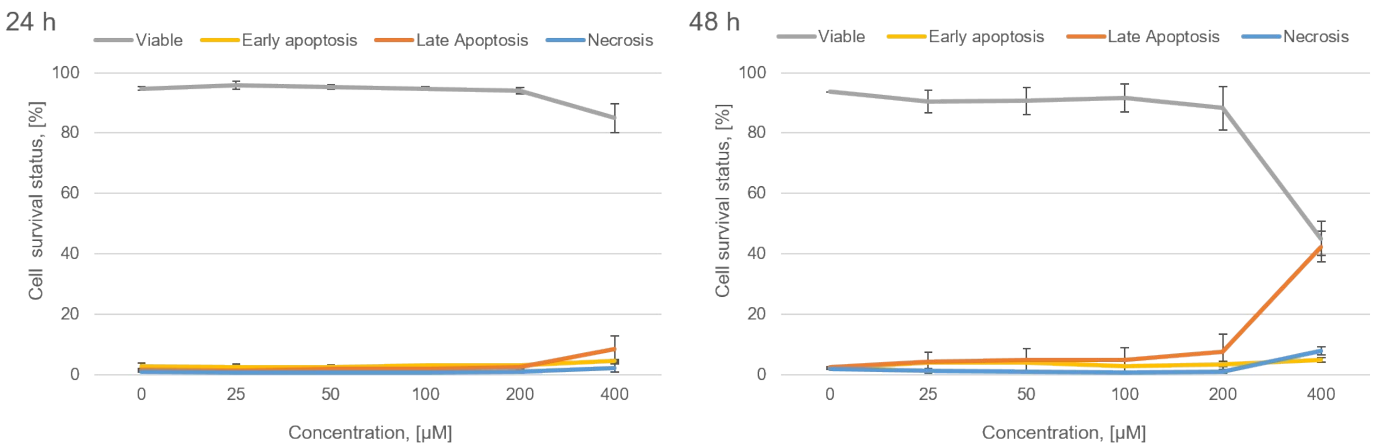

[C], µM	Viable cells	Early apoptosis	Late apoptosis	Necrosis
0	94.6 ± 0.6	2.8 ± 0.9	1.6 ± 0.5	0.9 ± 0.3
25	95.9 ± 1.2	2.3 ± 1.1	1.3 ± 0.5	0.5 ± 0.2
50	95.2 ± 0.6	2.5 ± 0.6	1.7 ± 0.9	0.6 ± 0.2
100	94.7 ± 0.7	2.9 ± 0.4	1.7 ± 0.9	0.7 ± 0.2
200	94 ± 1	2.9 ± 0.6	2.3 ± 0.7	0.8 ± 0.2
400	85 ± 4.8	4.5 ± 0.6	8.5 ± 4.1	2.1 ± 1.3

[C], µM	Viable cells	Early apoptosis	Late apoptosis	Necrosis
0	93.6 ± 0.1	2.1 ± 0.6	2.3 ± 0.1	1.9 ± 0.4
25	90.5 ± 3.7	3.9 ± 0.3	4.3 ± 3.2	1.3 ± 0.8
50	90.6 ± 4.6	3.9 ± 0.3	4.8 ± 3.9	0.8 ± 0.3
100	91.6 ± 4.6	2.7 ± 0.3	4.9 ± 4.1	0.7 ± 0.3
200	88.2 ± 7.2	3.4 ± 0.9	7.5 ± 5.8	0.8 ± 0.4
400	45.1 ± 5.6	4.9 ± 0.8	42.3 ± 5.2	7.7 ± 1.3

Fig. S10. Apoptosis/necrosis induction in SW480 cells after 24 and 48 h exposure to oxaliplatin, measured by FACS using annexin V-FITC/propidium iodide double-staining. The experiment confirmed the late onset of oxaliplatin toxicity even at 400 µM resulting in less than 20% dead cells after 24 h exposure. The effect is clearly enhancing for the same concentration with the continuation of exposure (>50% dead cells in 48 h). The late-onset for cytotoxicity of platinum(II)-based chemotherapeutics was previously shown for cisplatin (Fig. S9 and Fig. S10 in Supporting Information of Legin et al., *Chem Sci*, 2014;5(8):3135-43).
